# Towards a tailored approach for patients with acute diverticulitis and abscess formation. The DivAbsc2023 multicentre case–control study

**DOI:** 10.1007/s00464-024-10793-z

**Published:** 2024-04-17

**Authors:** Mauro Podda, Marco Ceresoli, Marcello Di Martino, Monica Ortenzi, Gianluca Pellino, Francesco Pata, Benedetto Ielpo, Valentina Murzi, Andrea Balla, Pasquale Lepiane, Nicolo’ Tamini, Giulia De Carlo, Alessia Davolio, Salomone Di Saverio, Luca Cardinali, Emanuele Botteri, Nereo Vettoretto, Pier Paolo Gelera, Belinda De Simone, Antonella Grasso, Marco Clementi, Danilo Meloni, Gaetano Poillucci, Francesco Favi, Roberta Rizzo, Giulia Montori, Giuseppa Procida, Irene Recchia, Ferdinando Agresta, Francesco Virdis, Stefano Piero Bernardo Cioffi, Martina Pellegrini, Massimo Sartelli, Federico Coccolini, Fausto Catena, Adolfo Pisanu

**Affiliations:** 1 Department of Surgical Science, University of Cagliari, Cagliari, Italy; 2 General and Emergency Surgery Department, School of Medicine and Surgery, Milano-Bicocca University, Monza, Italy; 3 Department of Health Sciences, Università del Piemonte Orientale, Novara, Italy; 4 Department of General and Emergency Surgery, Polytechnic University of Marche, Ancona, Italy; 5 Department of Colorectal Surgery, Vall d’Hebron University Hospital, Universitat Autonoma de Barcelona UAB, Barcelona, Spain; 6 Department of Advanced Medical and Surgical Sciences, Universitá degli Studi della Campania ‘Luigi Vanvitelli’, Naples, Italy; 7 Department of Pharmacy, Health and Nutritional Sciences, University of Calabria, Rende, Italy; 8 Hepatobiliary Surgery Unit, Hospital del Mar, Barcelona, Spain; 9 Coloproctology and Inflammatory Bowel Disease Surgery Unit, IRCCS San Raffaele Scientific Institute, Milan, Italy; 10 General and Minimally Invasive Surgery, Hospital “San Paolo”, Civitavecchia, Rome, Italy; 11 Department of Surgery, Madonna del Soccorso Hospital, San Benedetto del Tronto, Italy; 12 General Surgery Unit, ASST Spedali Civili, Montichiari, Brescia, Italy; 13 Department of General and Metabolic Surgery, Poissy and Saint-Germain-en-Laye Hospitals, Poissy, France; 14 General Surgery Unit, San Salvatore Hospital, Department of Biotechnology and Applied Clinical Sciences, University of L’Aquila, L’Aquila, Italy; 15 Department of General, Minimally Invasive and Robotic Surgery, S. Matteo Degli Infermi Hospital, Spoleto, Perugia, Italy; 16 Department of General and Emergency Surgery, Bufalini Hospital, Cesena, Italy; 17 Department of General Surgery, ULSS2 Marca Trevigiana, Vittorio Veneto, Treviso, Italy; 18 Trauma and Acute Care Surgery Unit, “Niguarda Ca Granda” Hospital, Milan, Italy; 19 Department of Surgery, Macerata Civil Hospital, Macerata, Italy; 20 General, Emergency, and Trauma Surgery Unit, Pisa University Hospital, Pisa, Italy; 21 Emergency Surgery Unit, Department of Surgical Science, University of Cagliari, Policlinico Universitario “D. Casula”, Azienda Ospedaliero-Universitaria di Cagliari, SS 554, Km 4,500, 09042 Monserrato, Italy

**Keywords:** Diverticular abscess, Conservative treatment, Non-operative treatment, Failure, Risk factors, Percutaneous drainage

## Abstract

**Background:**

This multicentre case–control study aimed to identify risk factors associated with non-operative treatment failure for patients with CT scan Hinchey Ib-IIb and WSES Ib-IIa diverticular abscesses.

**Methods:**

This study included a cohort of adult patients experiencing their first episode of CT-diagnosed diverticular abscess, all of whom underwent initial non-operative treatment comprising either antibiotics alone or in combination with percutaneous drainage. The cohort was stratified based on the outcome of non-operative treatment, specifically identifying those who required emergency surgical intervention as cases of treatment failure. Multivariable logistic regression analysis to identify independent risk factors associated with the failure of non-operative treatment was employed.

**Results:**

Failure of conservative treatment occurred for 116 patients (27.04%). CT scan Hinchey classification IIb (aOR 2.54, 95%CI 1.61;4.01, *P* < 0.01), tobacco smoking (aOR 2.01, 95%CI 1.24;3.25, *P* < 0.01), and presence of air bubbles inside the abscess (aOR 1.59, 95%CI 1.00;2.52, *P* = 0.04) were independent predictors of failure. In the subgroup of patients with abscesses > 5 cm, percutaneous drainage was not associated with the risk of failure or success of the non-operative treatment (aOR 2.78, 95%CI − 0.66;3.70, *P* = 0.23).

**Conclusions:**

Non-operative treatment is generally effective for diverticular abscesses. Tobacco smoking's role as an independent risk factor for treatment failure underscores the need for targeted behavioural interventions in diverticular disease management. IIb Hinchey diverticulitis patients, particularly young smokers, require vigilant monitoring due to increased risks of treatment failure and septic progression. Further research into the efficacy of image-guided percutaneous drainage should involve randomized, multicentre studies focussing on homogeneous patient groups.

**Graphical abstract:**

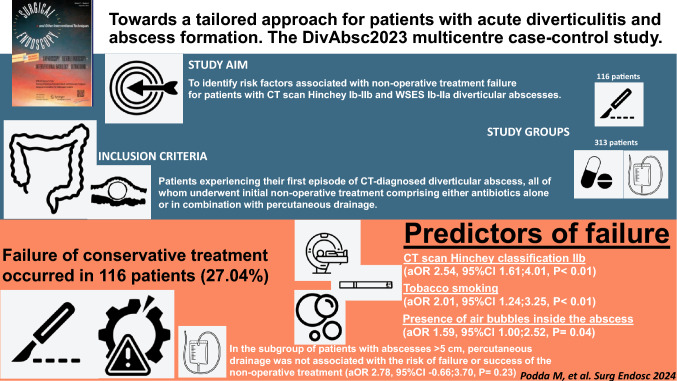

**Supplementary Information:**

The online version contains supplementary material available at 10.1007/s00464-024-10793-z.

Diverticular disease is a common clinical entity whose prevalence has risen steadily in Western countries over the past few decades [1]. It might lead to acute diverticulitis in approximately 4–7% of patients, among which 15–20% are complicated by an intra-abdominal abscess [2–4]. Complicated acute diverticulitis with abscess can be classified according to the original or the modified Hinchey classifications as type Ib (confined pericolic abscess smaller than 5 cm) or Hinchey II (pelvic, distant intra-abdominal or retroperitoneal abscess at least 5 cm in size) [4]. According to the World Society of Emergency Surgery (WSES) classification system, the Ib stage indicates the presence of an abscess ≤ 4 cm, while the IIa stage indicates an abscess > 4 cm [5]. Although there has been a wealth of research in the form of both observational and randomized studies focussing upon the treatment of Hinchey I, III, or IV disease stages [6–9], little focus has been placed upon the cohort of patients presenting with acute diverticulitis complicated by pericolic or pelvic abscesses. Abscess size of 4–6 cm is generally accepted as a reasonable cut-off determining the choice of treatment between antibiotic therapy and antibiotic therapy plus percutaneous drainage [5, 10–13]. Unfortunately, the available data to suggest this approach consists of a short list of studies with a limited number of patients, often associated with a short follow-up period, which is insufficient to assess the value of percutaneous drainage as the mainstay of treatment [14–16].

A subgroup of patients will fail the non-operative approach and require a surgical rescue strategy. Data from observational studies suggest an approximately 20–30% failure rate for both antibiotics with drainages and antibiotics alone [17, 18]. Several studies have explored the possible factors leading to the failure of the conservative approach [19, 20]. The identification of the subset of patients with risk predictors for non-operative treatment strategy failure is a clinically relevant, as well as unexplored field to date, the knowledge of which could improve decision-making processes, treatment strategies, patient counselling, and even modify the planned treatment pathway in patients deemed at highest risk of failure.

A multicentre retrospective cohort study was set up that merges the cases of acute complicated diverticulitis with abscess formation (CT scan Hinchey Ib and IIa/b, and WSES Ib/IIa) initially approached conservatively at twelve high-volume centres for emergency surgery in Italy. The aim was to assess the short-term outcomes of initial non-operative treatment for these patients and to identify risk factors associated with non-operative treatment failure to help facilitate appropriate patient selection and assess the optimal treatment strategy for this peculiar subgroup of patients.

## Patients and methods

This study complied with the Declaration of Helsinki principles and was waived by the central Italian Ethics Committee due to its retrospective, observational design. Informed consent was waived according to the regulations of the Italian Ethics Committee for retrospective analyses. Institutional review board approval was obtained at each participating centre. Reporting was performed according to the requirements of the STROBE (STrengthening the Reporting of Observational studies in Epidemiology) checklist [21]. The study protocol was registered in ClinicalTrials.gov (NCT06109506).

### Clinical data

Electronic medical records were reviewed retrospectively for patient demographic and baseline characteristics, comorbidity, clinical variables, and biochemistry markers related to the initial presentation of the disease, abscess size, location, the interval between the occurrence of symptoms and treatment, complications, and clinical outcomes after non-operative treatment. Comorbidities were scored using the Charlson Comorbidity Index (CCI) [22]. The reports of the CT scans were analysed for terms like diverticular abscess, pericolic free air, maximum diameter of the abscess, or covered perforation. Whenever missing information from the CT scan notes was reported, a second radiologist reviewed images, and missing data were retrieved. Pericolic air was defined as air located less than 5 cm from the affected segment of the colon, regardless of whether the air was intra or retroperitoneal. Free air was defined as extraluminal air 5 cm or more from the site of inflammation or in contact with the anterior peritoneal wall in the supine position [20, 23, 24]. Abscess measurements were performed, including the maximal area in the axial plane and the maximal diameter in the axial, coronal, and sagittal planes.

### Inclusion criteria

The patient cohort consisted of adult (≥ 18 years of age) patients who had CT-diagnosed complicated diverticulitis of the left colon with abscess formation (CT scan Hinchey Ib-IIb and WSES Ib-IIa diverticular abscesses, with or without localized pericolic extraluminal air) and who received initial non-operative treatment, being either antibiotics alone or antibiotics with percutaneous drainage of the abscess. For multiple recurrent cases for the same patient, only the first case treated was included in the database. The decision to focus solely on patients experiencing their first episode of acute diverticulitis with abscess formation was informed by a thorough review of the literature, according to which the number of prior episodes of acute diverticulitis predicts severity, with patients presenting for the first time having more severe disease than those with recurrent presentations [25–28].

To be included in the present study, patients had to show haemodynamic stability at hospital admission and no clinical or radiological evidence of diffuse peritonitis.

### Exclusion criteria

Patients were excluded from this study if they had perforated diverticulitis with diffuse (CT scan Hinchey III or IV, WSES stage IV) abdominal fluid at the CT scan or clinically assessed generalised peritonitis. Patients who received emergency surgery within 24 h after presentation were also excluded. Patients with a final diagnosis other than diverticular abscess, including those diagnosed with colonic cancer mimicking acute diverticulitis with abscess intraoperatively or after routine follow-up, were also excluded.

### Definition of outcomes

The primary study endpoint was the occurrence of non-operative treatment failure, defined as failure whenever a patient required emergency surgery or died due to abdominal sepsis during the hospital stay despite the planned non-operative treatment. The non-operative treatment was defined as successful when the acute infective condition was resolved conservatively, with the regression of symptoms and the normalization of inflammatory indexes, and the patient was discharged without the need for surgical intervention. Clinical definitions of non-operative treatment failure included at least one of the following: the occurrence of diffuse peritonitis, septic shock, increased size of the abscess at the follow-up CT scan, presence of four-quadrant free abdominal fluid at the follow-up CT scan, or lack of dimensional improvement of the abscess at the follow-up CT scan. Follow-up CT scans were performed during the index hospital admission. In all cases, in order to define the failure of conservative therapy, patient's clinical deterioration had to be documented.

Patients with planned colorectal resection for complicated acute diverticulitis after the success of non-operative treatment were excluded. Any adverse events after the discharge date (readmission, radiologic interventions, surgical intervention, and mortality) were not considered failures of non-operative treatment.

The secondary outcome was the recurrence of acute diverticulitis requiring hospital readmission within 90 days after discharge (within 30 days or after 30 days from the index episode). Admissions for elective operations were excluded from this outcome. In-hospital morbidity (infections, surgical complications, adverse drug reactions) was graded based on severity according to the Clavien-Dindo classification, based on the number of patients with at least one complication [29]. Although policies for percutaneous drainage placement may differ from centre to centre, guidelines for establishing the correct indication of the procedure were followed in the participating sites [5, 10].

After the initial attempt of conservative treatment with antibiotics plus/minus percutaneous drainage, if the patient required emergency surgery, a consultant-level on-call surgeon decided to operate based on the clinical condition, laboratory parameters, and radiological findings suggestive of patient deterioration, clinical worsening, or not improving conditions. The reasons for the decision to operate were registered in the clinical notes and translated into the database. The > 30 days patient follow-up was conducted by contacting the patients via telephone and obtaining all the pertinent clinical notes on eventual further episodes of recurrent sigmoid diverticulitis requiring hospital admission after the initial non-operative approach.

### Statistics

Statistical analysis was performed using SPSS® Statistics version 26 (IBM, Armonk, NY, USA). Descriptive and inferential statistics were provided for all previously mentioned variables. Distribution was examined by the Shapiro–Wilk test. Continuous variables were presented as mean or median values with standard deviation (or interquartile range IQR) with differences between groups assessed by the Student's *T*-test or Mann–Whitney *U* test. Patients were categorized based on the presence or absence of non-operative treatment failure. Categorical variables were presented in frequency tables, and statistical differences between groups were determined through Chi-square linear-by-linear association, Pearson *X*^*2*^*,* or Fisher exact test.

Differences in patient baseline and disease characteristics between subjects with and without non-operative treatment failure were assessed to identify risk factors for the outcomes. Univariable logistic regression analyses were performed to calculate crude odds ratios (ORs) with 95% confidence intervals (95% CI). Independent variables reaching a cut-off *P* value of < 0.05 at univariable analysis were considered for inclusion in multivariable logistic regression models. Continuous variables were transformed into dichotomic variables to provide a clear cut-off value by implementing the Youden index.

Multivariable logistic regression models under different methods (backward, forward, stepwise selection) were created to determine the independent risk factors for failure of non-operative treatment and recurrence. A *P* value < 0.05 was considered statistically significant. Multiple imputation and Maximum Likelihood Estimation (MLE) methods were implemented to impute missing data, avoid selection bias, and assess the potential impact of missing data on the results. Data were assumed to be missing at random. Model goodness-of-fit was evaluated, and multicollinearity among variables was assessed. Sensitivity analyses restricting the analysis to cases of abscess > 5 cm and CT scan Hinchey IIb stage diverticulitis were implemented.

## Results

A search for the International Classification of Diseases, 9th revision, codes 562.11, 562.13, 567.22, 567.38, 569.5 (acute diverticular disease of the bowel) was performed in the electronic patient files. The query for 2018–2022 produced 5,834 patients treated for diverticular disease at the twelve participating hospitals. Patients diagnosed with acute diverticulitis of the left colon were selected. Screening of electronic patient records identified 2.884 patients with CT scan-verified acute colonic diverticulitis. Of these, 480 had CT-verified Hinchey Ib or Hinchey IIa/b diverticular abscesses according to the classifications by Wasvary et al*.* [30], Sher et al*.* [31], and WSES (World Society of Emergency Surgery) Ib-IIa acute diverticulitis [5].

**Fig. 1:**
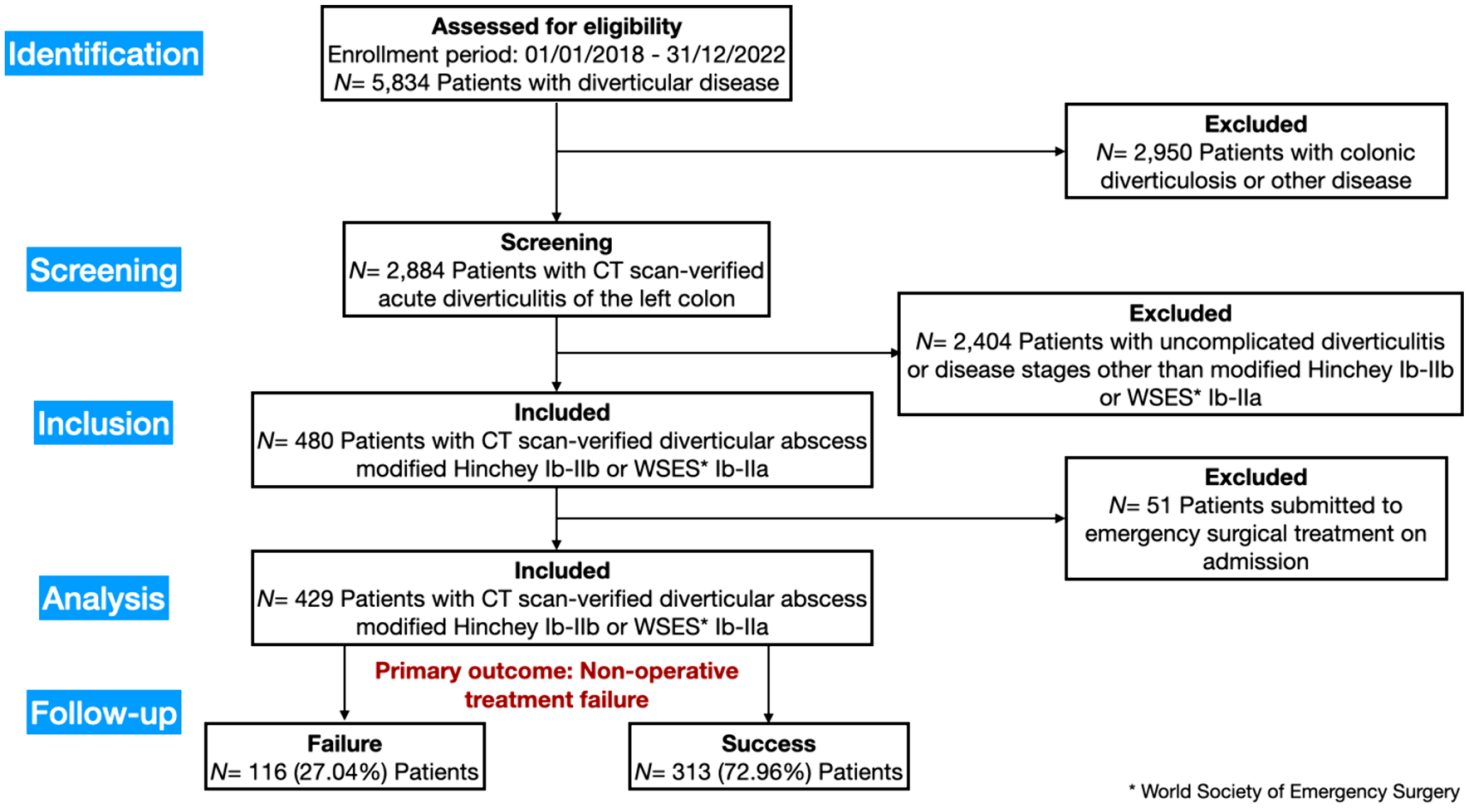
STROBE flow diagram of the study enrollment process

Fifty-one patients with diverticular abscesses were submitted to emergency surgical treatment on admission, and they were excluded from the present analysis; 429 patients with diverticular abscesses treated conservatively were finally included in this study (Fig. 1).

### Study population, imaging characteristics, and disease-specific characteristics

Patient and disease characteristics of the study cohort and the modality of non-operative treatment (antibiotics alone or antibiotics and percutaneous drainage) are shown in Table 1. Twenty-six (6.07%) were CT scan Hinchey Ib, 226 (52.68%) were CT scan Hinchey IIa, and 171 (39.86%) were CT scan Hinchey IIb patients. The mean abscess diameter on the CT scan was 41.17 mm (± 25.97). Abscesses were further stratified into the following three groups: < 3 cm (196 patients, 45.68%), 3–5 cm (124 patients, 28.90%), and > 5 cm (109 patients, 25.30%) to obtain more homogeneous samples for further sensitivity analyses.

**Table 1:** Baseline characteristics and modality of non-surgical management of the patients who underwent non-operative treatment with antibiotics and antibiotics plus percutaneous drainage

Variable mean ± SD or *N*. (%)	Missing data	Results	
Age (years) Mean ± SD	0	61.46 ± 14.02; 95%CI 60.69–60.13	
Body Mass Index (BMI) (Kg/m^2^)	0	26.81 ± 3.79; 95%CI 27.17–26.45	
Charlson Comorbidity Index	0	2.02 ± 2.22; 95%CI 2.23–1.81	
White Blood Cells (WBC) × 10^3^/μl	0	13.63 ± 4.28; 95%CI 14.04–13.23	
CRP (C-reactive Protein) (mg/l)	0	114.30 ± 79.53; 95%CI 121.83–106.78	
Creatinine (mg/dl)	47	1.010 ± 0.44; 95%CI 1.05–0.97	
Hemoglobin (mg/l)	47	13.00 ± 1.68; 95%CI 13.87–12.83	
Platelets × 10^3^/μl	48	269.92 ± 90.12; 95%CI 278.97–260.87	
Procalcitonin ng/ml	372	3.62 (1.39 IQR); 95%CI 7.35–0.09	
Body temperature (°C)	0	37.29 ± 0.93; 95%CI 37.38–37.20	
Systolic blood pressure (mmHg)	140	132.82 ± 19.24; 95%CI 135.04–130.60	
Heart rate	148	83.51 ± 16.67; 95%CI 85.46–81.56	
Abscess diameter (maximum) on CT scan (mm)	21	41.17 ± 25.97; 95%CI 43.69–38.65	
Time between the start of the antibiotic therapy and failure (days)	313	6.28 ± 6.03; 95%CI 7.38–5.19	
Time between the beginning of symptoms and the hospital admission	0	3.46 ± 2.69; 95%CI 3.71–3.20	
Time spent in the Emergency Department (minutes)	160	433.66 (291.10 IQR); 95%CI 526.82–340.51	
Number of previous episodes of acute diverticulitis	0	No previous episodes	278 (64.80%)
		1 previous episode	107 (24.94%)
		> 1 previous episodes	44 (10.26%)
Number of abscesses on CT scan	0	1 abscess	386 (89.98%)
		2 abscesses	36 (8.39%)
		> 2 abscesses	7 (1.63%)
Hinchey CT scan classification (modified by Sher et al*.* and Wasvary et al*.*)	0	Hinchey Ib	26 (6.06%)
		Hinchey IIa	226 (52.68%)
		Hinchey IIb	177 (41.26%)
Number of air bubbles inside the abscess	0	No bubble	271 (63.17%)
		1 bubble	80 (20.51%)
		> 1 bubble	70 (16.32%)
Time of hospital admission	96	06.01–12.00	101 (23.54%)
		12.01–18.00	94 (21.91%)
		18.01–23.59	94 (21.91%)
		00.00–06.00	44 (10.26%)
N. of cases treated/year	0	≤ 10/year	115 (26.8%)
		> 10/year	314 (73.2%)
Immunodeficiency (Congenital/Acquired)	0	5 (1.16%)	
Diabetes	0	39 (9.09%)	
Chronic Kidney Disease	0	19 (4.42%)	
Dialysis	0	2 (0.46%)	
Leukopenia	0	0 (0%)	
Active tumor	0	3 (0.69%)	
AIDS	0	0 (0%)	
Steroid therapy	0	17 (3.96%)	
Chemotherapy	0	2 (0.46%)	
Immunotherapy	0	3 (0.69%)	
Chronic cardiac failure	0	13 (3.03%)	
Chronic pulmonary failure	0	6 (1.39%)	
Obesity	0	69 (16.08%)	
Coagulopathy	0	5 (1.16%)	
High blood pressure (hypertension)	0	168 (39.16%)	
Chronic Obstructive Pulmonary Disease (COPD)	0	20 (4.66%)	
Tobacco smoking	0	112 (26.10%)	
Alcohol abuse	0	15 (3.49%)	
Clostridium Difficile infection	0	2 (0.46%)	
Abscess diameter < 3 cm	0	196 (45.68%)	
Abscess diameter 3–5 cm	0	124 (28.90%)	
Abscess diameter > 5 cm	0	109 (25.30%)	
World Society of Emergency Surgery (WSES) CT scan Ib	0	244 (56.87%)	
World Society of Emergency Surgery (WSES) CT scan IIa	0	185 (43.12%)	
Previous episodes of acute diverticulitis	0	No previous episodes	278 (64.80%)
		1 previous episode	107 (24.94%)
		> 1 previous episode	44 (10.25%)
Presence of retroperitoneal bubbles	0	13 (3.03%)	
Presence of distant free air	0	44 (10.25%)	
Presence of free pelvic fluid	0	134 (21.23%)	
CT-guided percutaneous drainage	0	27 (6.29%)	
Ultrasound-guided percutaneous drainage	0	24 (5.59%)	

Most patients (378 patients, 88.12%) were initially treated with antibiotics alone; 27 patients (6.29%) and 24 patients (5.59%) were submitted to CT-guided and Ultrasound-guided percutaneous drainage, respectively.

### Clinical outcomes

Failure of conservative treatment occurred for 116 patients (27.04%) (Table 2). A mortality rate of 0.69% was observed, corresponding to three patients. Detailed analysis revealed one fatality attributed to pneumonia, while the remaining two fatalities occurred subsequent to unsuccessful rescue surgeries, performed due to the progression of sepsis in the wake of non-operative treatment failure.

**Table 2:** Clinical outcomes of the patients who underwent non-operative treatment with antibiotics and antibiotics plus percutaneous drainage

Variable Mean ± SD or *N*. (%)	Missing data	Results	
Length of antibiotic therapy (days)	204	8.73 ± 3.68; 95%CI 9.21–8.25	
Length of hospital stay (days)	3	11.29 ± 7.34; 95%CI 11.99–10.60	
In-hospital morbidity	0	No morbidity	360 (86.25%)
		Clavien-Dindo I	19 (4.43%)
		Clavien-Dindo II	20 (4.66%)
		Clavien-Dindo IIIa	7 (1.63%)
		Clavien-Dindo IIIb	12 (2.80%)
		Clavien-Dindo IVa	1 (0.23%)
		Clavien-Dindo IVb	–
Symptomatic acute diverticulitis recurrence ≤ 30 days	0	5 (1.16%)	
Symptomatic acute diverticulitis recurrence > 30 days (to 90-day follow-up)	0	30 (6.99%)	
Characteristics of the diverticulitis recurrence (if any)	0	No recurrence	394
		Obstruction	4 (11.42%)
		Abscess	19 (54.29%)
		Perforation	12 (34.29%)
Failure of the conservative treatment	0	116 (27.04%)	
Reason for failure	313	Acute diffuse peritonitis	44 (37.93%)
		Septic shock	13 (11.20%)
		Increased size of the abscess at the follow-up CT scan	23 (19.82%)
		Four quadrant free abdominal fluid at the follow-up CT scan	2 (1.72%)
		Lack of dimensional improvement at the follow-up CT scan	34 (29.33%)
In-hospital mortality	0	3 (0.69%)	
Treatment of the failure: Laparoscopic lavage	0	16 (13.79%)	
Treatment of the failure: Hartmann resection	0	42 (36.20%)	
Treatment of the failure: Colorectal resection with primary anastomosis	0	58 (50.0%)	
Treatment of the failure: Colorectal resection with open abdomen	0	10 (8.62%)	

The mean duration of hospital stay was 11.29 days (± 7.34), with a difference between the patients who experienced failure of the non-operative treatment and those with success (16.67 ± 9.79 vs 9.30 ± 4.88, *P* < 0.01). In-hospital morbidity was more frequent in the failure group than in the success group, with most complications classified as Clavien-Dindo I-IIIa in the general analyses (Supplementary Table 1). A total of 116 patients required emergency surgery due to non-operative treatment failure and clinical deterioration (mean time between the start of the antibiotic therapy and failure 6.28 ± 6.03 days), with a total of 126 surgical operations registered. The indication for emergency surgery was the occurrence of diffuse acute peritonitis during conservative treatment (37.93%), septic shock (11.20%), increased size of the abscess at the follow-up CT scan (19.82%), presence of four-quadrant free abdominal fluid at the follow-up CT scan (1.72%), and lack of dimensional improvement of the abscess at the follow-up CT scan accompanied by clinical deterioration (29.33%).

After discharge, early recurrence (within 30 days from the discharge) was detected in five cases (1.16%), whereas symptomatic recurrence of acute diverticulitis beyond 30 days was reported for 30 patients (6.99%).

### Correlation analyses

#### Risk factors for conservative treatment failure and emergency surgery

**Fig. 2:**
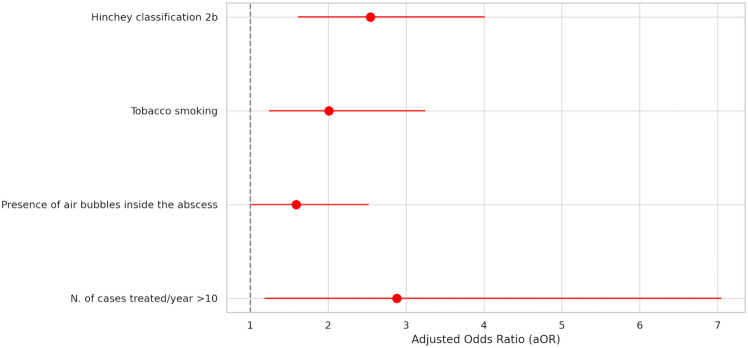
Multivariable analysis of risk factors for conservative treatment failure (general population)

Results of the univariable analysis are reported in Supplementary Table 1. The results of the multivariable logistic regression analysis of non-operative treatment failure are summarized in Supplementary Table 2 and Fig. 2. Within the stepwise models, CT scan Hinchey classification IIb (aOR 3.62, 95%CI 2.00;5.56, P < 0.01), tobacco smoking (aOR 2.72, 95%CI 1.45;5.11, *P* < 0.01), WBC count > 15 (× 10^3^ μ/l) (aOR 1.86, 95%CI 1.01;3.41, *P* = 0.04), and presence of air bubbles inside the abscess (aOR 1.82, 95%CI 1.00;3.13, *P* = 0.04) were predictive of non-operative treatment failure. These findings were confirmed in the conventional model, where CT scan Hinchey classification IIb (aOR 2.54, 95%CI 1.61;4.01, *P* < 0.01), tobacco smoking (aOR 2.01, 95%CI 1.24;3.25, *P* < 0.01), presence of air bubbles inside the abscess (aOR 1.59, 95%CI 1.00;2.52, *P* = 0.04), and centres treating more than 10 cases per year (aOR 2.88, 95%CI 1.18;7.05, *P* = 0.02) resulted to be independent predictors of failure of the non-operative treatment.

### Risk factors for recurrence of acute diverticulitis during the follow-up

A phone call follow-up was performed in 308 cases. Five patients in the study population (1.16%) experienced an early recurrence of symptomatic acute diverticulitis and a second hospitalisation within 30 days from hospital discharge. Results of the univariable and multivariable analyses are summarized in Supplementary Table 3. Thirty patients in the study population (6.99%) experienced a late recurrence of symptomatic acute diverticulitis and a second hospitalisation beyond 30 days (to 90-day follow-up) from hospital discharge. Regarding late recurrence after 30 days from hospital discharge, at the multivariable analysis, the number of previous episodes of acute diverticulitis increased the risk of recurrence and re-hospitalisation: previous episodes in general (aOR 11.04, 95%CI 1.92;3.87, *P* < 0.01), one previous episode (aOR 5.79, 95%CI 1.77;2.74, *P* < 0.01) and more than one previous episode (aOR 7.04, 95%CI 1.76;3.13, *P* = 0.01) were the most relevant independent predictors of late recurrence (Supplementary Table 4).

### Sensitivity analyses

#### Abscess > 5 cm

**Fig. 3:**
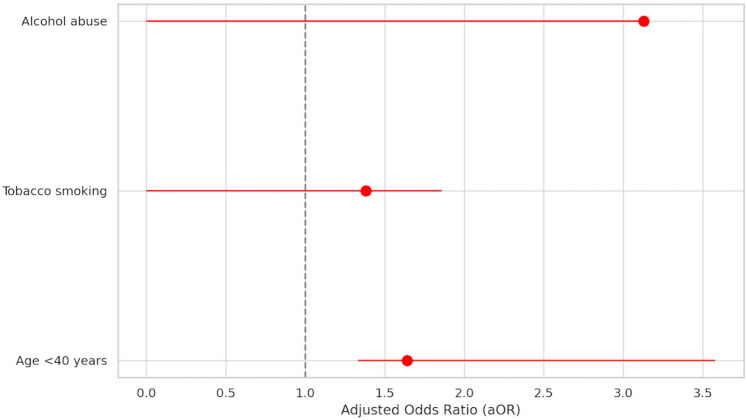
Multivariable analysis of risk factors for conservative treatment failure (subgroup: abscesses > 5 cm)

The analysis of predictive risk factors for non-operative treatment failure in the subgroup of patients with abscesses > 5 cm at CT scan is shown in Supplementary Tables 5 and 6. In this subgroup of patients, 44 (40.74%) experienced failure of non-operative treatment. The results of the multivariable logistic regression analysis of non-operative treatment failure are shown in Fig. 3. Within the stepwise models, age < 40 years (aOR 1.18, 95%CI 1.05;3.32, *P* = 0.04) was the only independent risk predictor of non-operative treatment failure. This result was confirmed in the conventional model (aOR 1.64, 95%CI 1.33;3.58, *P* = 0.01). Tobacco smoking showed a minor positive prediction role within one of the stepwise models (aOR 1.35, 95%CI 1.14;1.85, *P* = 0.04).

#### CT scan Hinchey IIb stage

The analysis of predictive risk factors for non-operative treatment failure in the subgroup of patients with CT scan Hinchey IIb stage disease is shown in Supplementary Tables 7 and 8. In this subgroup of patients, 67 (39.18%) experienced failure of non-operative treatment.

**Fig. 4:**
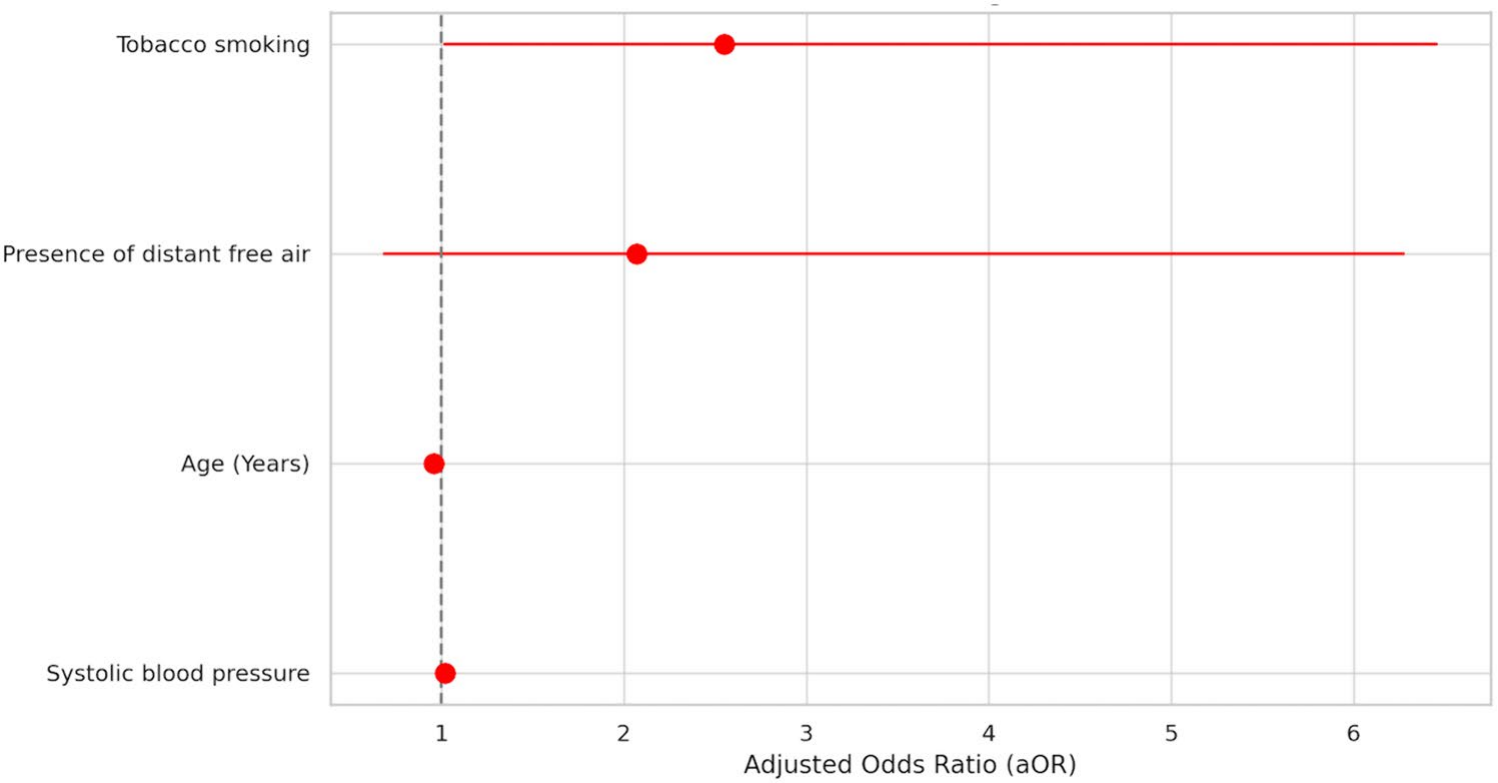
Multivariable analysis of risk factors for conservative treatment failure (subgroup: CT scan Hinchey IIb)

The results of the multivariable logistic regression analysis of non-operative treatment failure are summarized in Fig. 4. Within the stepwise models, age < 40 years (aOR 1.29, 95%CI 1.07;1.85, *P* = 0.02), tobacco smoking (aOR 2.75, 95%CI 1.10;6.47, *P* = 0.04) and systolic blood pressure (aOR 1.02, 95%CI 1.00;1.05, *P* = 0.02) were independent risk predictors of non-operative treatment failure. The conventional multivariable model confirmed that tobacco smoking (aOR 2.55, 95%CI 1.01;6.46, *P* = 0.04) and systolic blood pressure (aOR 1.02, 95%CI 1.01;1.04, *P* = 0.04) were predictive factors of non-operative treatment failure.

### The role of percutaneous drainage

Image-guided percutaneous drainage was placed in 51 patients (11.88%), of which 27 patients (6.29%) were CT-guided, and 24 patients (5.59%) were Ultrasound-guided. In the subset of patients with abscesses > 5 cm at CT scan, percutaneous drainage was not associated with the outcome of non-operative treatment (aOR 2.78, 95%CI − 0.66;3.70, *P* = 0.23) and symptomatic recurrence of acute diverticulitis beyond 30 days from the discharge (aOR 2.36, 95%CI − 4.45;4.49, *P* = 0.99). Percutaneous drainage showed no significant impact on the length of hospital stay (aOR 1.00, 95%CI − 0.15;1.17, *P* = 0.92), and the length of antibiotic therapy (aOR 1.01, 95%CI − 0.23;1.26, *P* = 0.90) (Table 3).

**Table 3:** Multivariable analysis of the role of percutaneous drainage for patients with diverticular abscesses > 5 cm on clinical outcomes (failure of non-operative treatment, length of antibiotic therapy, length of hospital stay, and recurrence of acute diverticulitis)

Variable	Adjusted Odds Ratio (aOR)	*P* value	95% confidence interval (CI)
Abscesses > 5 cm			
Failure of the non-operative treatment	2.78	0.23	− 0.66;3.70
Length of antibiotic therapy	1.01	0.90	− 0.23;1.26
Length of hospital stay	1.00	0.92	− 0.15;1.17
Symptomatic acute diverticulitis recurrence ≤ 30 days	2.77	0.99	− 7.73;7.77
Symptomatic acute diverticulitis recurrence > 30 days (to 90-day follow-up)	2.36	0.99	− 4.45;4.49

### Missing data

Among all candidate predictors in the multivariable models, only heart rate (148 missing values, 34.49%) and systolic blood pressure (140 missing values, 32.63%) had missing data. Sensitivity analyses of the non-imputed data showed similar results for the primary and secondary outcomes.

## Discussion

Over the years, treatment strategies for acute diverticulitis with abscess formation have gradually shifted from acute surgery to non-operative management comprised of antibiotic therapy with or without percutaneous drainage. A recent systematic review and meta-analysis by Fowler et al*.* [32] found that the failure rates following non-operative management of diverticular abscess did not significantly decrease over the past three decades. Within this context, identifying risk factors for failure can indicate which patients' careful supervision is necessary to avoid further complications through a septic progression of the disease.

This multicentre observational study conducted in twelve high-volume Italian surgical units for emergency surgery indicates that non-operative treatment with antibiotics alone or with the placement of a CT- or Ultrasound-guided percutaneous drainage is an effective strategy in avoiding emergency surgery for patients with diverticular abscess. The results of our study showed success rates of 72.96% in the general population of patients with diverticular abscess, in line with the results of previous studies [17, 33].

Previous studies suggest that patients presenting for the first time typically exhibit more severe disease manifestations than those with recurrent episodes [25, 26]. Additionally, the literature indicates that complications such as perforation or complicated diverticulitis occur more frequently in patients during their initial episode compared to subsequent occurrences [27, 28]. Given this evidence, we believe that our study's focus on the initial episode of acute diverticulitis with abscess formation allows for a more precise assessment of the condition's severity and the effectiveness of the treatment approaches under investigation.

Despite lacking high-quality evidence, international guidelines recommend percutaneous drainage according to abscess size [5, 10, 34]. A diverticular abscess diameter of 5 cm is generally accepted as a cut-off determining whether percutaneous drainage is suggested in addition to antibiotics [5]. Our findings reflect a percutaneous drainage rate appearing low (11.88%) compared with the range reported in the literature, which varies from 24 to 48% [33, 35] for CT scan Hinchey II abscesses. This variability can largely be attributed to the nature of the evidence guiding these recommendations, which stems from small-scale, non-randomised studies. The lack of large, definitive randomised controlled trials in this area has led to considerable heterogeneity in clinical practice, as reflected in our study population.

In our study, percutaneous drainage combined with antibiotics as a treatment for abscesses larger than 5 cm did not offer clear advantages in the short- and long-term success of the non-operative treatment compared with only antibiotics. Some published studies have corroborated these outcomes. Ocaña et al*.* [35] showed that the modality of initial non-operative management did not appear to be independently associated with treatment failure and emergency surgery. However, in their study, in a stratified analysis by initial treatment and abscess size, a higher risk of emergency surgery was reported in the antibiotics alone group compared with percutaneous drainage in patients with ≥ 6 cm abscesses. Elagili et al*.* [13] found that 21% of treatments with antibiotics alone failed as initial treatment compared with 18% of the percutaneous group, without statistical difference. Similarly, Mali et al*.* [33] showed an equivalent non-operative treatment failure rate between antibiotics and percutaneous drainage in a cohort of 241 patients with abscesses of 4 cm or more. Lambrichts et al*.* [19], in a large cohort of 447 patients, showed a significantly higher rate of conservative treatment failure and subsequent emergency surgery in the percutaneous drainage group (13.9%) than in the antibiotics alone group (7.2%). In the present study, the advantageous role of positioning a percutaneous drain in decreasing the failure rate of non-operative treatment and recurrences of acute diverticulitis was not demonstrated, both in the general population and in patients with abscesses larger than 5 cm in size.

Confounding by indication of initial treatment cannot be ruled out from our analysis, and the differences could primarily reflect disease, comorbidity, and clinical severity at admission. Our study, however, confirmed that for abscesses larger than 5 cm, it can still be debated which treatment strategy is most appropriate. To research the potential role of percutaneous drainage, its probable impact on the length of hospital stay and the duration of antibiotic therapy was explored. No association with these outcomes was observed on multivariable analysis. These results, which must be interpreted within the limitations related to the retrospective study design and, therefore, the potential selection bias resulting from it, call for the need to investigate more in detail the role of image-guided percutaneous drainage in patients with diverticular abscess through the design of possibly randomised, multicentre studies with large series of homogeneous populations.

The recurrence rate in our study was in line with those reported in the recent literature (10–55%) [36]. This wide range of recurrence rates may be attributed to varying lengths of follow-up, as longer follow-up is associated with increasing recurrence rate [37]. Al-Masrouri et al*.* [38] reported the incidence of readmission for treatment failure in the general population with acute diverticulitis to be 6.6% at 90-day follow-up. However, they reached 12.5% among patients with an index presentation of complicated diverticulitis, and an index episode of complicated diverticulitis was the strongest risk factor for treatment failure.

It seems clear that the decision to treat patients with diverticular abscesses conservatively should not be based solely on abscess size or CT scan Hinchey stage, but other patient and disease characteristics should be considered. The role of age in the evolution of complicated diverticulitis, especially in the subgroup of patients with abscesses larger than 5 cm, was highlighted by our study. The data showed how young age was an independent risk factor for the failure of non-operative treatment, although these results may be affected by a selection bias. Indeed, despite the pre-specified criteria for the definition of conservative treatment failure provided in the study protocol, the threshold for establishing the failure of non-operative treatment and subsequent emergency surgery in younger patients may have been lower than in older patients.

Last, from the results of our study comes the need to emphasize the impact of some habits, including tobacco smoking, on the probability of failure of non-operative treatment. Several studies have investigated the association between smoking and acute diverticulitis. Our data are in line with those from published studies, which demonstrated that tobacco smoking is associated with a higher risk of conservative treatment failure in patients with diverticular abscesses [39–41]. Recently, a single-centre cohort study by Murzi et al*.* on 71 patients with CT scan Hinchey II diverticular abscess found that tobacco smoking was the only independent predictor of conservative treatment failure [18]. Tobacco smoking can be a pathogenetic and prognostic factor through several mechanisms. Firstly, nicotine acts by inhibiting cytokines (IL-1 and TNF) and leukocytes, reducing collagen formation, causing an increase in intraluminal pressure with impaired blood flow to the colonic mucosa and increasing tissue permeability. Such mechanisms can favour the evolution towards perforation and increase inflammation, favouring endothelial dysfunction and reducing the blood oxygen supply [42–44].

Patients with Hinchey stage IIb disease have a relevant risk of failure of the non-operative strategy, as reported in previous studies [32], and they should probably be considered for upfront operative management. Notably, in the general multivariable analysis of our study, CT scan Hinchey IIb disease stage and tobacco smoking were shown to have the same prognostic impact on the failure of non-operative therapy, the first with an aOR of 2.54, and the second with an aOR of 2.01.

Our experience has shown how, even in the subgroup of patients with CT scan Hinchey IIb stage, age < 40 years and tobacco smoking were the strongest independent factors predictive of failure of non-operative treatment. This aligns with previous reports where patients with larger abscesses and more advanced stages of diverticulitis may have a higher likelihood of treatment failure and require surgical intervention [5]. While the effectiveness and predominant role of non-operative therapy with antibiotics alone, or antibiotics and CT- or Ultrasound-guided percutaneous drainage in patients with diverticular abscess is recognised, the results of this study allow for the focus on predictive risk factors of failure of the non-operative treatment, primarily tobacco smoking, to be shifted. In light of our findings, it becomes evident that the success of non-operative treatment for diverticular abscesses is not solely contingent on disease-specific characteristics but also significantly influenced by patient-related factors, notably lifestyle habits such as tobacco smoking. This insight underscores the necessity of incorporating smoking status into the clinical decision-making process when evaluating the suitability of non-operative treatment for this patient subgroup. The augmented risk of non-operative treatment failure and septic progression in patients with stage IIb Hinchey diverticulitis, especially among younger individuals who smoke, advocates a more cautious, personalized treatment strategy that considers smoking status as a critical factor.

Furthermore, our results focus on some subgroups of patients, such as young people under 40 years of age and patients with CT scan Hinchey stage IIb diverticulitis, for whom close follow-up is required after the start of non-operative treatment, in order to identify the clinical and laboratory signs of failure of the conservative strategy as early as possible, to be able to quickly implement all surgical rescue measures and prevent the appearance of fatal complications of abdominal sepsis [45].

While this large multicentre retrospective cohort study offers valuable insights into various aspects of non-operative treatment strategies of patients with diverticular abscess, it also comes with several inevitable limitations associated with a retrospective study design and the characteristics of the practice at the participating centres, which introduces the potential for selection bias and confounding by indication. An inherent limitation of our study pertains to the threshold for establishing failure of non-operative treatment and the subsequent decision to proceed with emergency surgery. Despite our efforts to delineate precise criteria for the definition of treatment failure, we acknowledge that the ultimate decision to transition from non-operative to surgical management is influenced by individual surgeon behaviour and clinical judgment. This variability reflects the complex, multifaceted nature of clinical decision-making in acute diverticulitis cases, where surgeons must weigh the specific clinical presentation, patient preferences, and evolving standards of care. Moreover, variability in healthcare practices, patient populations, and data collection methods among different centres can have introduced heterogeneity between hospital differences in treatment, firstly regarding the reasons for choosing percutaneous drainage, potentially affecting the study's external validity. Another possible bias is the potential risk of missing data, as the availability of variables and outcomes data depends on the completeness of medical records. Within this context, multiple imputation methods were used to prevent selection bias introduced by missing data for certain variables. Despite these limitations, this large multicentre retrospective cohort study can provide valuable insights into treatment patterns and the occurrence of a rare event, such as the failure of non-operative treatment of diverticular abscess.

In conclusion, a non-operative approach is successful in most patients with diverticular abscesses. The success of non-operative treatment is dependent not only on disease-specific characteristics but also on patient-related factors, including the individual patient's habits, clinical condition, and response to treatment. The role of tobacco smoking as an independent predictor of failure of non-operative therapy of acute diverticulitis complicated by diverticular abscesses reported in this large multicentre study highlights the importance of prevention.

It is crucial to monitor patients with stage IIb CT scan Hinchey diverticulitis undergoing non-operative treatment with caution due to the augmented risk of non-operative treatment failure and septic progression in this subgroup of patients, especially if they are young and tobacco smokers. The results of this study could improve surgical decision-making in these patients, although prospective studies need to confirm the external validity of our findings.

### Supplementary Information

Below is the link to the electronic supplementary material.

Supplementary file1 (DOCX 29 kb)

Supplementary file2 (DOC 133 kb)

Supplementary file3 (DOC 34 kb)

Supplementary file4 (DOC 47 kb)

Supplementary file5 (DOC 51 kb)

Supplementary file6 (DOC 112 kb)

Supplementary file7 (DOC 31 kb)

Supplementary file8 (DOC 117 kb)

Supplementary file9 (DOC 29 kb)

## Data Availability

All information is freely available by application to the Chief Investigator Mauro Podda (Department of Surgical Science, University of Cagliari).
