# Supplementary material for: Towards a tailored approach for patients with acute diverticulitis and abscess formation. The DivAbsc2023 multicentre case–control study
Source: Surg Endosc. 2024 Apr 17;38(6):3180–94. doi: 10.1007/s00464-024-10793-z (PMC11133057; doi:10.1007/s00464-024-10793-z)
Supplement: Supplementary file 2 — Supplementary file2 (DOC 133 kb) [file 464_2024_10793_MOESM2_ESM.doc]

**Supplementary Table 1.** Results of the univariable analysis of risk factors for conservative treatment failure (General Population).

| ***Variable*** | ***Missing data*** | ***Failure*** | ***Success*** | ***P value*** | ***Odds Ratio (OR) or Mean Difference (MD and Effect size (SE)*** | ***95% Confidence Interval (CI)*** |
| --- | --- | --- | --- | --- | --- | --- |
| ***Failure of conservative treatment*** | ***0*** | ***116 (27.04%)*** | ***313 (72.96%)*** |  |  |  |
| **Age (years)** | 0 | 59.30 ± 15.02 | 62.26 ± 13.57 | 0.05 | 2.96 (1.51) | 0.02;5.94 |
| **Body Mass Index (BMI) (Kg/m^2^)** | 0 | 26.55 ± 4.16 | 26.90 ± 3.64 | 0.39 | 0.34 (0.41) | -0.46;1.16 |
| **Charlson Comorbidity Index** | 0 | 2.05 ± 2.04 | 2.00 ± 2.28 | 0.84 | -0.04 (0.24) | -0.52;0.42 |
| **White Blood Cells (WBC) (x10^3^ u/l)** | 0 | 14.56 ± 4.28 | 13.28 ± 4.14 | <0.01 | -1.27 (0.46) | -2.18;-0.37 |
| **C-reactive Protein (CRP) mg/l** | 0 | 126.42 ± 4.14 | 109.81 ± 4.28 | 0.03 | -16.61 (8.61) | -33.55;-0.32 |
| **Creatinine (mg/dl)** | 47 | 1.01 ± 0.42 | 1.01 ± 0.45 | 0.95 | -0.03 (0.05) | -0.10;0.09 |
| **Hemoglobin (g/dl)** | 47 | 12.92 ± 1.72 | 13.3 ± 1.66 | 0.58 | 0.10 (0.19) | -0.27;0.49 |
| **Platelets (x10^3^ u/l)** | 48 | 282.14 ± 109.17 | 265.74 ± 82.41 | 0.12 | -16.40 (10.57) | -37.20;4.39 |
| **Procalcitonin (ng/ml)** | 372 | 0.98 (IQR 1.46) | 0.20 (IQR 1.10) | 0.06 | 2.86 (4.17) | -5.49;11.22 |
| **Body temperature (^o^C)** | 0 | 37.51 ± 0.93 | 37.20 ± 0.91 | <0.01 | -0.08 (0.05) | -0.50;-0.11 |
| **Systolic blood pressure (mmHg)** | 140 | 134.64 ± 19.52 | 132.14 ± 18.45 | 0.32 | -2.49 (2.55) | -7.51;2.52 |
| **Heart rate (bpm)** | 148 | 88.46 ± 16.55 | 81.70 ± 16.09 | <0.01 | -6.75 (2.21) | -11.12;-2.39 |
| **Abscess diameter on CT scan (mm)** | 21 | 44.50 (IQR 28.00) | 30.50 (IQR 26.55) | <0.01 | -12.83 (2.83) | -18.39;-7.27 |
| **Length of antibiotic therapy (days)** | 204 | 9.21 ± 2.94 | 8.57 ± 5.34 | 0.25 | -0.64 (0.57) | -1.77;0.47 |
| **Time between the beginning of symptoms and hospital admission (days)** | 0 | 3.80 ± 2.90 | 3.32 ± 2.59 | 0.10 | -0.47 (0.29) | -1.04;0.10 |
| **Time spent in the Emergency Department (minutes)** | 160 | 326.00 (IQR 290.75) | 300.00 (IQR 290.00) | 0.62 | 51.18 (104.50) | -154.57;256.94 |
| **Length of hospital stay (days)** | 3 | 16.67 ± 9.79 | 9.30 ± 4.88 | <0.01 | -7.37 (0.71) | -8.78;-5.96 |
| **Previous episodes of acute diverticulitis** | 0 | No previous episodes 74 (63.79%) | No previous episodes 204 (65.17%) | 0.63 | 0.03 (0.30) | -0.55;1.62 |
|  |  | 1 previous episode 27 (23.27%) | 1 previous episode 80 (25.55%) |  |  |  |
|  |  | > 1 previous episode 15 (12.93%) | > 1 previous episode 29 (9.26%) |  |  |  |
| **Number of abscesses on CT scan** | 0 | 1 abscess 109 (93.96%) | 1 abscess 277 (88.49%) | 0.09 | -1.15 (1.01) | -3.14;1.83 |
|  |  | 2 abscesses 7 (6.03%) | 2 abscesses 29 (9.26%) |  |  |  |
|  |  | >2 abscesses 0 (0%) | >2 abscesses 7 (2.23%) |  |  |  |
| **Hinchey classification on CT scan** | 6 | Hinchey Ib 4 (3.44%) | Hinchey Ib 22 (7.02%) | <0.001 | -0.89 (0.63) | -2.14;0.35 |
|  |  | Hinchey IIa 45 (38.79%) | Hinchey IIa 181 (57.82%) |  |  |  |
|  |  | Hinchey IIb 67 (57.75%) | Hinchey IIb 104 (33.22%) |  |  |  |
| **Air bubbles inside the abscess** | 0 | 0 bubbles 59 (50.86%) | 0 bubbles 212 (67.73%) | <0.01 | 0.41 (0.28) | -0.13;0.96 |
|  |  | 1 bubble 30 (25.86%) | 1 bubble 58 (18.53%) |  |  |  |
|  |  | >1 bubble 27 (23.27%) | >1 bubble 43 (13.73%) |  |  |  |
| **Time of hospital admission** | 96 | 06.01-12.00 36 (31.03%) | 06.01-12.00 65 (20.76%) | 0.14 | NA | NA |
|  |  | 12.01-18.00 31 (26.72%) | 12.01-18.00 63 (20.12%) |  |  |  |
|  |  | 18.01-23.59 20 (17.24%) | 18.01-23.59 74 (23.64%) |  |  |  |
|  |  | 00.00-06.00 14 (12.06%) | 00.00-06.00 30 (9.85%) |  |  |  |
| **In-hospital morbidity (Clavien-Dindo)** | 0 | No morbidity  66 (56.89%) | No morbidity  304 (97.12%) | <0.01 | 1.41 (0.29) | 1.84;2.99 |
|  |  | Clavien-Dindo 1  14 (12.06%) | Clavien-Dindo 1  5 (1.59%) |  |  |  |
|  |  | Clavien-Dindo 2  19 (16.37%) | Clavien-Dindo 2  1 (0.31%) |  |  |  |
|  |  | Clavien-Dindo 3a  4 (3.44%) | Clavien-Dindo 3a  3 (0.95%) |  |  |  |
|  |  | Clavien-Dindo 3b  12 (10.34%) | Clavien-Dindo 3b  0 (0.00%) |  |  |  |
|  |  | Clavien-Dindo 4a  1 (0.86%) | Clavien-Dindo 4a  0 (0.00%) |  |  |  |
|  |  | Clavien-Dindo 4b  0 (0%) | Clavien-Dindo 4b  0 (0%) |  |  |  |
| **Diverticulitis recurrence characteristics** | 0 | Obstruction 0 (0%) | Obstruction 4 (14.28%) | 0.54 | NA | NA |
|  |  | Abscess 4 (57.14%) | Abscess 15 (53.57%) |  |  |  |
|  |  | Perforation 3 (42.85%) | Perforation 9 (32.14%) |  |  |  |
| **Age >60 years** **(Youden J: 0.55)** | 0 | 57 (49.13%) | 179 (57.18%) | 0.13 | 0.72 | 0.47;1.10 |
| **Female gender** | 0 | 54 (46.55%) | 149 (47.60%) | 0.84 | 0.95 | 0.62;1.46 |
| **Body Mass Index (BMI) >28 Kg/m ^2^**  **(Youden J: 0.64)** | 0 | 33 (28.44%) | 107 (34.18%) | 0.26 | 0.76 | 0.48;1.22 |
| **Charlson Comorbidity Index >3**  **(Youden J: 0.56)** | 0 | 24 (20.69%) | 56 (17.89%) | 0.50 | 1.19 | 0.70;2.04 |
| **Immunodeficiency (Congenital/Acquired)** | 0 | 0 (0.00%) | 5 (1.59%) | 0.17 | 0.24 | 0.01;4.38 |
| **Diabetes** | 0 | 10 (8.62%) | 29 (9.26%) | 0.83 | 0.92 | 0.43;1.96 |
| **Chronic Kidney Disease** | 0 | 4 (3.44%) | 15 (4.79%) | 0.54 | 0.71 | 0.23;2.18 |
| **Dialysis** | 0 | 1 (0.86%) | 1 (0.31%) | 0.46 | 2.71 | 0.16;43.73 |
| **Leukopenia** | 0 | 0 (0.00%) | 0 (0.00%) | NA | NA | NA |
| **Active tumor** | 0 | 1 (0.86%) | 2 (0.63%) | 0.80 | 1.35 | 0.12;15.05 |
| **AIDS** | 0 | 0 (0.00%) | 0 (0.00%) | NA | NA | NA |
| **Steroid therapy** | 0 | 6 (5.17%) | 11 (3.51%) | 0.43 | 1.49 | 0.54;4.19 |
| **Chemotherapy** | 0 | 1 (0.86%) | 1 (0.31%) | 0.46 | 2.71 | 0.16;43.73 |
| **Immunotherapy** | 0 | 0 (0.00%) | 3 (0.95%) | 1.12 | 0.38 | 0.02;7.42 |
| **Chronic cardiac failure** | 0 | 5 (4.31%) | 8 (2.55%) | 0.34 | 1.71 | 0.55;5.36 |
| **Chronic pulmonary failure** | 0 | 1 (0.86%) | 5 (1.59%) | 0.56 | 0.53 | 0.06;4.63 |
| **Obesity** | 0 | 26 (22.41) | 43 (13.73%) | 0.03 | 1.81 | 1.05;3.12 |
| **Coagulopathy** | 0 | 2 (1.72%) | 3 (0.95%) | 0.51 | 1.81 | 0.29;10.98 |
| **High blood pressure (hypertension)** | 0 | 50 (43.10%) | 118 (37.70%) | 0.30 | 1.25 | 0.81;1.93 |
| **Chronic obstructive pulmonary disease (COPD)** | 0 | 5 (4.31%) | 15 (4.79%) | 0.83 | 0.89 | 0.31;2.52 |
| **Chronic ischemic heart disease** | 0 | 8 (6.89%) | 13 (4.15%) | 0.24 | 1.70 | 0.68;4.22 |
| **Tobacco smoking** | 0 | 41 (35.34%) | 71 (22.68%) | <0.01 | 1.86 | 1.17;2.96 |
| **Alcohol abuse** | 0 | 6 (5.17%) | 9 (2.87%) | 0.25 | 1.84 | 0.64;5.29 |
| **White Blood Cells (WBC) >15 x10 3/ul** **(Youden J: 0.68)** | 0 | 49 (42.24%) | 89 (28.43%) | <0.01 | 1.84 | 1.18;2.86 |
| **C-reactive Protein (CRP) >120 mg/l**  **(Youden J: 0.73)** | 0 | 52 (44.82%) | 104 (33.27%) | 0.02 | 1.63 | 1.05;2.52 |
| **Creatinine >1.6 mg/dl**  **(Youden J: 0.64)** | 47 | 8 (8.16%) | 17 (5.98%) | 0.45 | 1.39 | 0.58;3.34 |
| **Hemoglobin <13 g/dl**  **(Youden J: 0.64)** | 46 | 50 (51.02%) | 136 (47.88%) | 0.59 | 1.13 | 0.71;1.79 |
| **Platelets < 250 x10 3/ul**  **(Youden J: 0.93)** | 48 | 41 (42.26%) | 136 (47.88%) | 0.33 | 0.79 | 0.50;1.26 |
| **Body temperature >38 ^o^C**  **(Youden J: 0.98)** | 0 | 54 (46.55%) | 120 (38.33%) | 0.12 | 1.40 | 0.91;2.15 |
| **Heart rate >90 bpm**  **(Youden J: 0.65)** | 148 | 26 (34.66%) | 46 (22.33%) | 0.03 | 1.84 | 1.03;3.28 |
| **N. of cases treated/year >10** | 0 | 127 (40.4%) | 187 (59.6%) | <0.01 | 2.01 | 1.25;3.25 |
| **Clostridium Difficile infection** | 0 | 1 (0.86%) | 1 (0.31%) | 0.46 | 2.71 | 0.16;43.73 |
| **Abscess diameter <3 cm** | 0 | 32 (27.58%) | 164 (52.39%) | <0.01 | 0.34 | 0.21;0.55 |
| **Abscess diameter 3-5 cm** | 0 | 41 (35.34%) | 83 (26.51%) | 0.07 | 1.51 | 0.96;2.39 |
| **Abscess diameter >5 cm** | 0 | 44 (37.93%) | 64 (20.45%) | <0.01 | 2.36 | 1.48;3.77 |
| **World Society of Emergency Surgery (WSES) CT scan Ib** | 0 | 39 (33.62%) | 205 (65.49%) | <0.01 | 0.26 | 0.17;0.41 |
| **World Society of Emergency Surgery (WSES) CT scan IIa** | 0 | 77 (66.37%) | 108 (34.50%) | <0.01 | 3.74 | 2.38;5.87 |
| **Presence of air bubbles inside the abscess** | 0 | 54 (46.55%) | 99 (31.62%) | <0.01 | 1.88 | 1.21;2.91 |
| **Previous episodes of diverticulitis >1** | 0 | 15 (12.93%) | 29 (9.26%) | 0.62 | -0.12 | -0.62;0.37 |
| **Hinchey CT scan classification stage Ib** | 0 | 4 (3.44%) | 23 (7.32%) | 0.16 | 0.47 | 0.15;1.39 |
| **Hinchey CT scan classification stage IIa** | 0 | 45 (38.79%) | 181 (57.82%) | <0.01 | 0.46 | 0.29;0.71 |
| **Hinchey CT scan classification stage IIb** | 0 | 67 (57.75%) | 104 (33.22%) | <0.01 | 2.74 | 1.77;4.25 |
| **Presence of retroperitoneal bubbles** | 0 | 6 (5.17%) | 7 (2.23%) | 0.11 | 2.38 | 0.78;7.25 |
| **Presence of distant free air** | 0 | 23 (19.82%) | 21 (6.70%) | <0.01 | 3.43 | 1.82;6.49 |
| **Presence of free pelvic fluid** | 0 | 34 (29.31%) | 100 (31.94%) | 0.60 | 0.88 | 0.55;1.40 |
| **CT-guided percutaneous drainage** | 0 | 10 (8.62%) | 17 (5.43%) | 0.22 | 1.64 | 0.72;3.70 |
| **Ultrasound-guided percutaneous drainage** | 0 | 6 (5.17%) | 18 (5.75%) | 0.81 | 0.89 | 0.34;2.31 |
| **In-hospital mortality** | 0 | 2 (1.72%) | 1 (0.31%) | 0.12 | 5.47 | 0.49;60.94 |
| **Treatment of the failure: Laparoscopic lavage** | 0 | 16 (13.79%) | NA | NA | NA | NA |
| **Treatment of the failure: Hartmann resection** | 0 | 42 (36.20%) | NA | NA | NA | NA |
| **Treatment of the failure: Colorectal resection with primary anastomosis** | 0 | 58 (50.00%) | NA | NA | NA | NA |
| **Treatment of the failure: Colorectal resection with open abdomen** | 0 | 10 (8.62%) | NA | NA | NA | NA |
| **Time between the beginning of the symptoms and hospital admission >4 days** | 0 | 30 (25.86%) | 61 (19.48%) | 0.15 | 1.44 | 0.87;2.37 |
| **Day of hospital admission (weekend)** | 0 | 21 (18.10%) | 41 (13.09%) | 0.34 | 1.32 | 0.73;2.37 |
| **Symptomatic acute diverticulitis recurrence ≤30 days** | 0 | 2 (1.72%) | 3 (0.95%) | 0.51 | 1.81 | 0.29;10.98 |
| **Symptomatic acute diverticulitis recurrence >30 days (to 90-day follow-up**) | 0 | 5 (4.31%) | 25 (7.98%) | 0.18 | 0.51 | 0.19;1.38 |
