# Supplementary material for: Towards a tailored approach for patients with acute diverticulitis and abscess formation. The DivAbsc2023 multicentre case–control study
Source: Surg Endosc. 2024 Apr 17;38(6):3180–94. doi: 10.1007/s00464-024-10793-z (PMC11133057; doi:10.1007/s00464-024-10793-z)
Supplement: Supplementary file 3 — Supplementary file3 (DOC 34 kb) [file 464_2024_10793_MOESM3_ESM.doc]

**Supplementary Table 2.** Results of the multivariable analysis of risk factors for conservative treatment failure (General Population).

| ***Variable*** | ***Estimate*** | ***Standard Error*** | | ***Adjusted Odds Ratio (aOR)*** | | ***P Value*** | ***95% Confidence Interval (CI)*** | |
| --- | --- | --- | --- | --- | --- | --- | --- | --- |
| ***Model 1 Stepwise*** | | | | | | | | |
| Hinchey classification 2b | 1.68 | 0.66 | | 5.36 | | 0.01 | 1.46;19.68 | |
| Tobacco smoking | 1.01 | 0.32 | | 2.75 | | 0.01 | 1.44;5.26 | |
| Body temperature (C ^o^) | 0.39 | 0.16 | | 1.47 | | 0.01 | 1.07;2.02 | |
| Model Summary | McFadden R^2^ = 0.13; Negelkerke R^2^ = 0.21; Tjur R^2^ = 0.16; Cox&Snell R^2^ = 0.14 | | | | | | | |
| ***Model 2 Stepwise*** | | | | | | | | |
| Hinchey classification 2b | 1.58 | 0.67 | | 4.87 | | 0.01 | 1.30;18.24 | |
| Tobacco smoking | 1.03 | 0.33 | | 2.82 | | 0.01 | 1.46:5.41 | |
| Body temperature (C ^o^) | 0.39 | 0.16 | | 1.47 | | 0.01 | 1.07;2.02 | |
| Heart rate | 0.01 | 0.01 | | 1.01 | | 0.03 | 1.00;1.03 | |
| Model Summary | McFadden R^2^ = 0.15; Negelkerke R^2^ = 0.23; Tjur R^2^ = 0.17; Cox&Snell R^2^ = 0.16 | | | | | | | |
| ***Model 3 Stepwise*** | | | | | | | | |
| Hinchey classification 2b | 1.58 | 0.67 | | 4.87 | | 0.01 | 1.30;18.24 | |
| Tobacco smoking | 1.03 | 0.33 | | 2.82 | | 0.01 | 1.46;5.41 | |
| Body temperature (C ^o^) | 0.34 | 0.16 | | 1.40 | | 0.03 | 1.01;1.93 | |
| Heart rate | 0.01 | 0.01 | | 1.01 | | 0.05 | 1.00;1.03 | |
| Presence of air bubbles inside the abscess | 0.51 | 0.31 | | 1.67 | | 0.09 | 0.91;3.07 | |
| Model Summary | McFadden R^2^ = 0.15; Negelkerke R^2^ = 0.24; Tjur R^2^ = 0.18; Cox&Snell R^2^ = 0.16 | | | | | | | |
| ***Model 4 Stepwise*** | | | | | | | | |
| Hinchey classification 2b | 1.40 | 0.29 | | 4.07 | | <0.01 | 2.27;7.28 | |
| Tobacco smoking | 0.98 | 0.31 | | 2.67 | | <0.01 | 1.43;4.98 | |
| White Blood Cells (WBC) >15 x10^3^ u/l | 0.72 | 0.30 | | 2.06 | | 0.01 | 1.13;3.73 | |
| Model Summary | McFadden R^2^ = 0.12; Negelkerke R^2^ = 0.20; Tjur R^2^ = 0.15; Cox&Snell R^2^ = 0.13 | | | | | | | |
| ***Model 5 Stepwise*** | | | | | | | | |
| Hinchey classification 2b | 1.28 | 0.30 | | 3.62 | | <0.01 | 2.00;5.56 | |
| Tobacco smoking | 1.00 | 0.32 | | 2.72 | | 0.02 | 1.45;5.11 | |
| White Blood Cells (WBC) >15 x10^3^ u/l | 0.62 | 0.30 | | 1.86 | | 0.04 | 1.01;3.41 | |
| Presence of air bubbles inside the abscess | 0.60 | 0.305 | | 1.82 | | 0.04 | 1.00;3.13 | |
| Model Summary | McFadden R^2^ = 0.14; Negelkerke R^2^ = 0.22; Tjur R^2^ = 0.15; Cox&Snell R^2^ = 0.15 | | | | | | | |
| ***Model 6 Enter*** | | | | | | | | |
| Hinchey classification 2b | 0.93 | 0.23 | | 2.54 | | <0.01 | 1.61;4.01 | |
| Tobacco smoking | 0.69 | 0.24 | | 2.01 | | <0.01 | 1.24;3.25 | |
| Presence of air bubbles inside the abscess | 0.46 | 0.23 | | 1.59 | | 0.04 | 1.00;2.52 | |
| N. of cases treated/year >10 | 1.06 | 0.45 | | 2.88 | | 0.02 | 1.18;7.05 | |
| **Model Summary** | McFadden R^2^ = 0.06; Negelkerke R^2^ = 0.10; Tjur R^2^ = 0.07; Cox&Snell R^2^ = 0.07 | | | | | | | |
| **Accuracy** 0.76 | **AUC** 0.76 | | **Sensitivity** 0.36 | | **Specificity** 0.91 | | | **Precision** 0.61 |
