# Supplementary material for: Towards a tailored approach for patients with acute diverticulitis and abscess formation. The DivAbsc2023 multicentre case–control study
Source: Surg Endosc. 2024 Apr 17;38(6):3180–94. doi: 10.1007/s00464-024-10793-z (PMC11133057; doi:10.1007/s00464-024-10793-z)
Supplement: Supplementary file 4 — Supplementary file4 (DOC 47 kb) [file 464_2024_10793_MOESM4_ESM.doc]

**Supplementary Table 3.** Results of the univariable and multivariable analyses of risk factors for recurrence of acute diverticulitis ≤30 days (General Population).

| ***Variable*** | ***P value*** | ***Odds Ratio (OR) with 95%CI*** | ***P value*** | ***Adjusted Odds Ratio (OR) with 95%CI*** |
| --- | --- | --- | --- | --- |
| ***Recurrence of Acute Diverticulitis*** | 0.28 | 1.03 (-0.03;0.10) |  |  |
| **Age (years)** | 0.17 | 4.52 (-0.69;3.70) |  |  |
| **Body Mass Index (BMI) (Kg/m^2^)** | 0.28 | 1.12 (-0.09;0.32) |  |  |
| **Charlson Comorbidity Index** | 0.55 | 1.07 (-0.16;0.30) |  |  |
| **White Blood Cells (WBC) (x10^3^ u/l)** | 0.22 | 0.08 (-0.06;0.28) |  |  |
| **C-reactive Protein (CRP) mg/l** | 0.30 | 0.99 (-0.02;0.01) |  |  |
| **Creatinine (mg/dl)** | 0.61 | 0.42 (-4.18;2.47) |  |  |
| **Hemoglobin (g/dl)** | 0.89 | 0.96 (-0.56;0.48) |  |  |
| **Platelets (x10^3^ u/l)** | 0.56 | 1.00 (-0.01;0.01) |  |  |
| **Procalcitonin (ng/ml)** | 0.80 | 0.69 (-0.34;0.27) |  |  |
| **Body temperature (^o^C)** | 0.23 | 1.92 (-0.41;1.71) |  |  |
| **Systolic blood pressure (mmHg)** | 0.12 | 1.19 (-0.04;0.40) |  |  |
| **Heart rate (bpm)** | 0.15 | 1.08 (-0.03;0.19) |  |  |
| **Abscess diameter on CT scan (mm)** | 0.88 | 1.00 (-0.03;0.04) |  |  |
| **Length of antibiotic therapy (days)** | 0.12 | 0.70 (-0.78;0.09) |  |  |
| **Time between the beginning of symptoms and hospital admission (days)** | 0.18 | 0.56 (-1.41;0.27) |  |  |
| **Time spent in the Emergency Department (minutes)** | 0.11 | 0.98 (-0.03;0.01) |  |  |
| **Length of hospital stay (days)** | < 0.01 | 1.11 (0.04;0.16) | 0.01 | 1.09 (0.01;0.16) |
| **Previous episodes of acute diverticulitis** | 0.69 | 0.64 (-2.63;1.76) |  |  |
| **Number of abscesses on CT scan** | 0.99 | 2.42 (-3.52;3.49) |  |  |
| **Hinchey classification on CT scan** | 0.12 | 0.11 (-5.01;0.59) |  |  |
| **Air bubbles inside the abscess** | 0.47 | 0.44 (-3.00;1.39) |  |  |
| **Time of hospital admission** | 0.36 | 0.35 (-3.32;1.23) |  |  |
| **In-hospital morbidity (Clavien-Dindo)** | <0.01 | 20.44 (1.00;5.03) | 0.03 | 16.53 (1.19;5.41) |
| **Gender** | 0.17 | 4.52 (-0.69;3.70) |  |  |
| **Immunodeficiency (Congenital/Acquired)** | 0.99 | 1.97 (-3.48;3.45) |  |  |
| **Diabetes** | 0.41 | 2.53 (-1.28;3.14) |  |  |
| **Chronic Kidney Disease** | 0.99 | 2.57 (-4.85;4.82) |  |  |
| **Dialysis** | 0.99 | 5.39 (-3.33;3.31) |  |  |
| **Active tumor** | 0.99 | 1.97 (-4.48;4.46) |  |  |
| **Steroid therapy** | 0.10 | 6.37 (-0.39;4.10) |  |  |
| **Chemotherapy** | 0.99 | 5.39 (-3.33;3.31) |  |  |
| **Immunotherapy** | 0.99 | 1.97 (-4.48;4.46) |  |  |
| **Chronic cardiac failure** | 0.99 | 7.10 (-3.55;3.53) |  |  |
| **Chronic pulmonary failure** | 0.99 | 1.96 (-3.17;3.15) |  |  |
| **Obesity** | 0.16 | 3.55 (-0.54;3.07) |  |  |
| **Coagulopathy** | 0.99 | 1.97 (-3.48;3.45) |  |  |
| **High blood pressure (hypertension)** | 0.10 | 6.34 (-0.35;4.04) |  |  |
| **Chronic obstructive pulmonary disease (COPD)** | 0.99 | 2.57 (-4.72;4.69) |  |  |
| **Chronic ischemic heart disease** | 0.99 | 2.55 (-4.61;4.58) |  |  |
| **Tobacco smoking** | 0.75 | 0.70 (-2.55;1.85) |  |  |
| **Alcohol abuse** | 0.99 | 7.07 (-3.31;3.28) |  |  |
| **Clostridium Difficile infection** | 0.99 | 5.39 (-3.33;3.31) |  |  |
| **Abscess diameter <3 cm** | 0.16 | 4.83 (-0.62;3.77) |  |  |
| **Abscess diameter 3-5 cm** | 0.99 | 7.02 (-3.13;3.10) |  |  |
| **Abscess diameter >5 cm** | 0.78 | 0.73 (-2.50;1.89) |  |  |
| **World Society of Emergency Surgery (WSES) CT scan Ib** | 0.45 | 0.50 (-2.49;1.10) |  |  |
| **World Society of Emergency Surgery (WSES) CT scan IIa** | 0.45 | 1.99 (-1.10;2.49) |  |  |
| **Presence of air bubbles inside the abscess** | 0.47 | 0.44 (-3.00;1.39) |  |  |
| **Hinchey CT scan classification stage Ib** | 0.22 | 3.98 (-0.84;3.61) |  |  |
| **Hinchey CT scan classification stage IIa** | 0.17 | 0.22 (-3.70;0.69) |  |  |
| **Hinchey CT scan classification stage IIb** | 0.36 | 2.28 (-0.97;2.62) |  |  |
| **Presence of retroperitoneal bubbles** | 0.99 | 7.10 (-3.55;3.53) |  |  |
| **Presence of distant free air** | 0.48 | 2.21 (-1.41;3.00) |  |  |
| **Presence of free pelvic fluid** | 0.59 | 0.54 (-2.80;1.59) |  |  |
| **CT-guided percutaneous drainage** | 0.23 | 3.82 (-0.88;3.56) |  |  |
| **Ultrasound-guided percutaneous drainage** | 0.99 | 2.54 (-4.31;4.28) |  |  |
