# Supplementary material for: Towards a tailored approach for patients with acute diverticulitis and abscess formation. The DivAbsc2023 multicentre case–control study
Source: Surg Endosc. 2024 Apr 17;38(6):3180–94. doi: 10.1007/s00464-024-10793-z (PMC11133057; doi:10.1007/s00464-024-10793-z)
Supplement: Supplementary file 5 — Supplementary file5 (DOC 51 kb) [file 464_2024_10793_MOESM5_ESM.doc]

**Supplementary Table 4.** Results of the univariable and multivariable analyses of risk factors for recurrence of acute diverticulitis >30 days (General Population).

| ***Variable*** | ***P value*** | ***Odds Ratio (OR) with 95%CI*** | ***P value*** | ***Adjusted Odds Ratio (OR) with 95%CI*** |
| --- | --- | --- | --- | --- |
| ***Recurrence of Acute Diverticulitis*** |  |  |  |  |
| **Age (years)** | 0.31 | 0.98 (-0.04;0.01) |  |  |
| **Body Mass Index (BMI) (Kg/m^2^)** | 0.99 | 1.00 (-0.09;0.09) |  |  |
| **Charlson Comorbidity Index** | 0.21 | 0.85 (-0.39;0.08) |  |  |
| **White Blood Cells (WBC) (x10^3^ u/l)** | 0.34 | 0.95 (-0.13;0.04) |  |  |
| **C-reactive Protein (CRP) mg/l** | 0.73 | 0.99 (-0.06;0.04) |  |  |
| **Creatinine (mg/dl)** | 0.67 | 1.17 (-0.67;0.93) |  |  |
| **Hemoglobin (g/dl)** | 0.02 | 1.33 (0.03;0.54) | <0.01 | 1.83 (0.19;1.02) |
| **Hemoglobin <13 (g/dl)** **(Youden J: 0.54)** | 0.03 | 0.38 (-1.84;-0.05) | 0.03 | 0.37 (-1.93;-0.05) |
| **Platelets (x10^3^ u/l)** | 0.10 | 0.99 (-0.01;0.01) |  |  |
| **Procalcitonin (ng/ml)** | 0.90 | 0.99 (-0.10;0.09) |  |  |
| **Body temperature (^o^C)** | <0.01 | 1.81 (0.15;1.03) | 0.17 | 1.49 (-0.17;0.97) |
| **Body temperature >38 (^o^C)** **(Youden J: 0.55)** | 0.01 | 2.71 (1.23;1.77) | 0.11 | 2.01 (-0.16;1.56) |
| **Systolic blood pressure (mmHg)** | 0.01 | 0.96 (-0.06;-0.08) | 0.01 | 0.95 (-0.08;-0.09) |
| **Systolic blood pressure >135 (mmHg)**  **(Youden J: 0.62)** | 0.02 | 1.31 (1.21;1.86) | 0.56 | 0.75 (-1.22;0.67) |
| **Heart rate (bpm)** | 0.86 | 1.00 (-0.02;0.03) |  |  |
| **Abscess diameter on CT scan (mm)** | 0.47 | 1.00 (-0.08;0.01) |  |  |
| **Length of antibiotic therapy (days)** | 0.55 | 0.95 (-0.21;0.11) |  |  |
| **Time between the beginning of symptoms and hospital admission (days)** | 0.41 | 0.93 (-0.23;0.09) |  |  |
| **Time spent in the Emergency Department (minutes)** | 0.85 | 1.00 (-0.01;0.01) |  |  |
| **Length of hospital stay (days)** | 0.46 | 0.97 (-0.08;0.03) |  |  |
| **Previous episodes of acute diverticulitis** | <0.01 | 4.03 (0.55;2.24) | <0.01 | 11.04 (1.92;3.87) |
| **Number of previous episodes of acute diverticulitis = 1** | <0.01 | 2.87 (1.30;1.81) | <0.01 | 5.79 (1.77;2.74) |
| **Number of previous episodes of acute diverticulitis > 1** | 0.04 | 2.37 (1.19;1.82) | 0.01 | 7.04 (1.76;3.13) |
| **Number of abscesses on CT scan** | 0.46 | 2.21 (-1.35;2.94) |  |  |
| **Hinchey classification on CT scan** | 0.55 | 0.67 (-1.72;0.92) |  |  |
| **Air bubbles inside the abscess** | 0.36 | 1.41 (-0.40;1.09) |  |  |
| **Time of hospital admission** | 0.84 | 1.15 (-1.28;1.58) |  |  |
| **In-hospital morbidity (Clavien-Dindo)** | 0.16 | 2.48 (-0.38;2.20) |  |  |
| **Gender** | 0.11 | 0.53 (-1.41;0.15) |  |  |
| **Immunodeficiency (Congenital/Acquired)** | 0.28 | 3.40 (-0.99;3.44) |  |  |
| **Diabetes** | 0.63 | 0.69 (-1.83;1.11) |  |  |
| **Chronic Kidney Disease** | 0.54 | 1.60 (-1.04;1.98) |  |  |
| **Dialysis** | 0.06 | 13.72 (-0.17;5.41) |  |  |
| **Active tumor** | 0.98 | 2.29 (-1.65;1.63) |  |  |
| **Steroid therapy** | 0.09 | 3.05 (-0.19;2.42) |  |  |
| **Chemotherapy** | 0.99 | 2.29 (-2.03;2.00) |  |  |
| **Immunotherapy** | 0.12 | 6.84 (-0.50;4.35) |  |  |
| **Chronic cardiac failure** | 0.92 | 1.11 (-1.96;2.18) |  |  |
| **Chronic pulmonary failure** | 0.36 | 2.71 (-1.18;3.18) |  |  |
| **Obesity** | 0.16 | 0.35 (-2.49;0.42) |  |  |
| **Coagulopathy** | 0.99 | 8.39 (-2.11;2.08) |  |  |
| **High blood pressure (hypertension)** | 0.29 | 0.64 (-1.24;0.37) |  |  |
| **Chronic obstructive pulmonary disease (COPD)** | 0.59 | 1.51 (-1.09;1.92) |  |  |
| **Chronic ischemic heart disease** | 0.64 | 1.42 (-1.15;1.86) |  |  |
| **Tobacco smoking** | 0.72 | 0.85 (-1.03;0.71) |  |  |
| **Alcohol abuse** | 0.96 | 0.94 (-2.11;2.01) |  |  |
| **Clostridium Difficile infection** | 0.99 | 2.29 (-2.03;2.00) |  |  |
| **Abscess diameter <3 cm** | 0.78 | 0.90 (-0.85;0.64) |  |  |
| **Abscess diameter 3-5 cm** | 0.77 | 0.88 (-0.95;0.71) |  |  |
| **Abscess diameter >5 cm** | 0.80 | 0.89 (-0.98;0.76) |  |  |
| **World Society of Emergency Surgery (WSES) CT scan Ib** | 0.24 | 0.64 (-1.18;0.30) |  |  |
| **World Society of Emergency Surgery (WSES) CT scan IIa** | 0.24 | 1.55 (-0.30;1.18) |  |  |
| **Presence of air bubbles inside the abscess** | 0.36 | 1.41 (-0.40;1.09) |  |  |
| **Hinchey CT scan classification stage Ib** | 0.35 | 1.81 (-0.67;1.85) |  |  |
| **Hinchey CT scan classification stage IIa** | 0.29 | 0.66 (-1.15;0.34) |  |  |
| **Hinchey CT scan classification stage IIb** | 0.43 | 1.34 (-0.44;1.04) |  |  |
| **Presence of retroperitoneal bubbles** | 0.92 | 1.11 (-1.96;2.18) |  |  |
| **Presence of distant free air** | 0.50 | 0.60 (-1.96;0.97) |  |  |
| **Presence of free pelvic fluid** | 0.88 | 0.93 (-0.87;0.74) |  |  |
| **CT-guided percutaneous drainage** | 0.11 | 2.51 (-0.21;2.05) |  |  |
| **Ultrasound-guided percutaneous drainage** | 0.28 | 2.00 (-0.57;1.96) |  |  |
