# Supplementary material for: Towards a tailored approach for patients with acute diverticulitis and abscess formation. The DivAbsc2023 multicentre case–control study
Source: Surg Endosc. 2024 Apr 17;38(6):3180–94. doi: 10.1007/s00464-024-10793-z (PMC11133057; doi:10.1007/s00464-024-10793-z)
Supplement: Supplementary file 6 — Supplementary file6 (DOC 112 kb) [file 464_2024_10793_MOESM6_ESM.doc]

**Supplementary Table 5.** Results of the univariable analysis of risk factors for conservative treatment failure (Abscesses >5 cm).

| ***Variable*** | ***Missed data*** | ***Failure*** | ***Success*** | ***P value*** | ***Odds Ratio (OR), Mean Difference (SE)*** | ***95% Confidence Interval (CI)*** |
| --- | --- | --- | --- | --- | --- | --- |
| ***Conservative treatment*** |  | *44 (40.74%)* | *64 (59.26%)* |  |  |  |
| **Age (years)** | 0 | 57.40 ± 14.89 | 65.37 ± 14.06 | <0.01 | 7.96 (2.82) | 2.37;13.56 |
| **Body Mass Index (BMI) (Kg/m^2^)** | 0 | 26.52 ± 3.80 | 26.20 ± 4.02 | 0.67 | -0.32 (0.77) | -1.84;1.20 |
| **Charlson Comorbidity Index** | 0 | 1.84 ± 2.07 | 2.53 ± 2.02 | 0.08 | 0.69 (0.40) | -0.10;1.48 |
| **White Blood Cells (WBC) (x10^3^ u/l)** | 0 | 15.61 ± 4.23 | 14.00 ± 4.31 | 0.05 | -1.60 (0.83) | -3.26;0.05 |
| **C-reactive Protein (CRP) mg/l** | 0 | 138.85 ± 82.68 | 145.13 ± 98.67 | 0.73 | 6.27 (18.12) | -29.64;42.20 |
| **Creatinine (mg/dl)** | 6 | 0.97 ± 0.36 | 1.07 ± 0.70 | 0.41 | 0.09 (0.12) | -0.14;0.33 |
| **Hemoglobin (g/dl)** | 6 | 13.11 ± 1.86 | 12.90 ± 1.72 | 0.55 | -0.21 (0.36) | -0.93;0.50 |
| **Platelets (x10^3^ u/l)** | 6 | 306.53 ± 118.69 | 288.12 ± 102.08 | 0.40 | -18.41 (22.14) | -62.35;25.52 |
| **Procalcitonin (ng/ml)** | 81 | 1.22 ± 1.51 | 7.70 ± 23.89 | 0.48 | 6.48 (9.15) | -12.37;25.33 |
| **Body temperature (^o^C)** | 0 | 37.48 ± 0.89 | 37.45 ± 0.90 | 0.84 | -0.03 (0.17) | -0.38;0.31 |
| **Systolic blood pressure (mmHg)** | 34 | 134.20 ± 17.07 | 128.00 ± 19.41 | 0.15 | -6.20 (4.27) | -14.71;2.31 |
| **Heart rate (bpm)** | 36 | 93.47 ± 15.82 | 88.97 ± 19.89 | 0.29 | -4.49 (4.27) | -13.01;4.02 |
| **Abscess diameter on CT scan (mm)** | 0 | 77.36 ± 29.32 | 74.40 ± 19.08 | 0.52 | -2.95 (4.65) | -12.18;6.27 |
| **Length of antibiotic therapy (days)** | 64 | 6.89 ± 3.51 | 8.36 ± 2.89 | 0.13 | 1.46 (0.96) | -0.48;3.41 |
| **Time between the beginning of symptoms and hospital admission (days)** | 0 | 4.45 ± 3.64 | 4.37 ± 3.83 | 0.91 | -0.08 (0.73) | -1.53;1.37 |
| **Time spent in the Emergency Department (minutes)** | 35 | 395.41 ± 369.43 | 443.38 ± 372.81 | 0.58 | 47.93 (87.10) | -125.71;221.65 |
| **Length of hospital stay (days)** | 1 | 16.86 ± 8.44 | 11.56 ± 7.08 | <0.01 | -5.29 (1.510) | -8.29;-2.30 |
| **Previous episodes of acute diverticulitis** | 0 | No previous episodes  31 (70.45%) | No previous episodes  40 (62.50%) | 0.68 | -0.25 (0.31) | -0.86;0.35 |
|  |  | 1 previous episode  10 (22.72%) | 1 previous episode  18 (28.12%) |  |  |  |
|  |  | > 1 previous episode  3 (6.81%) | > 1 previous episode  6 (9.37%) |  |  |  |
| **Number of abscesses on CT scan** | 0 | 1 abscess  40 (90.09%) | 1 abscess  60 (93.75%) | 0.47 | 0.01 (0.18) | -0.35;0.38 |
|  |  | 2 abscesses  4 (9.09%) | 2 abscesses  3 (4.68%) |  |  |  |
|  |  | >2 abscesses  0 (0.00%) | >2 abscesses  1 (1.56%) |  |  |  |
| **Air bubbles inside the abscess** | 0 | 0 bubbles  19 (43.18%) | 0 bubbles  30 (46.87%) | 0.92 | 0.05 (0.21) | -0.36;0.47 |
|  |  | 1 bubble  13 (29.54%) | 1 bubble  17 (26.56%) |  |  |  |
|  |  | >1 bubble  12 (27.27%) | >1 bubble  17 (26.56%) |  |  |  |
| **In-hospital morbidity (Clavien-Dindo)** | 0 | No morbidity  25 (56.81%) | No morbidity  62 (96.87%) | <0.01 | 2.83 (0.52) | 1.81;3.85 |
|  |  | Clavien-Dindo 1  5 (11.36%) | Clavien-Dindo 1  1 (1.56%) |  |  |  |
|  |  | Clavien-Dindo 2  7 (15.90%) | Clavien-Dindo 2  0 (0.00%) |  |  |  |
|  |  | Clavien-Dindo 3a  1 (2.27%) | Clavien-Dindo 3a  1 (1.56%) |  |  |  |
|  |  | Clavien-Dindo 3b  5 (11.36%) | Clavien-Dindo 3b  0 (0.00%) |  |  |  |
|  |  | Clavien-Dindo 4a  1 (2.27%) | Clavien-Dindo 4a  0 (0.00%) |  |  |  |
|  |  | Clavien-Dindo 4b  0 (0.00%) | Clavien-Dindo 4b  0 (0.00%) |  |  |  |
| **Diverticulitis recurrence characteristics** | 0 | Obstruction  0 (0.00%) | Obstruction  1 (14.28%) | 0.38 |  |  |
|  |  | Abscess  0 (0.00%) | Abscess  4 (57.14%) |  |  |  |
|  |  | Perforation  1 (100.00%) | Perforation  2 (28.57%) |  |  |  |
| **Age >60 years** **(Youden J: 0.55)** | 0 | 21 (47.72%) | 41 (64.06%) | 0.09 | 0.51 | 0.23;1.11 |
| **Female gender** | 0 | 23 (52.27%) | 31 (48.43%) | 0.69 | 1.16 | 0.54;2.51 |
| **Body Mass Index (BMI) >28 Kg/m ^2^**  **(Youden J: 0.44)** | 0 | 12 (27.27%) | 20 (31.25%) | 0.93 | 1.03 | 0.43;2.46 |
| **Charlson Comorbidity Index >3**  **(Youden J: 0.64)** | 0 | 7 (15.90%) | 20 (31.25%) | 0.07 | 0.41 | 0.15;1.09 |
| **Immunodeficiency (Congenital/Acquired)** | 0 | 0 (0.00%) | 2 (3.12%) | 0.23 | 0.08 | 0.01;4.93 |
| **Diabetes** | 0 | 1 (2.27%) | 10 (15.62%) | 0.02 | 1.47 | 1.10;25.17 |
| **Chronic Kidney Disease** | 0 | 1 (2.27%) | 2 (3.12%) | 0.79 | 1.30 | 0.02;68.87 |
| **Active tumor** | 0 | 1 (2.27%) | 1 (1.56%) | 0.78 | 0.13 | 0.02;7.75 |
| **Steroid therapy** | 0 | 0 (0.00%) | 6 (9.37%) | 0.03 | 1.10 | 1.00;1.84 |
| **Chemotherapy** | 0 | 1 (2.27%) | 0 (0.00%) | 0.22 | 4.44 | 0.17;111.73 |
| **Immunotherapy** | 0 | 0 (0.00%) | 1 (1.56%) | 0.40 | 0.47 | 0.01;11.94 |
| **Chronic cardiac failure** | 0 | 2 (4.54%) | 2 (3.12%) | 0.70 | 1.47 | 0.20;10.89 |
| **Chronic pulmonary failure** | 0 | 1 (2.27%) | 2 (3.12%) | 0.79 | 0.72 | 0.06;8.20 |
| **Obesity** | 0 | 9 (20.45%) | 10 (15.62%) | 0.51 | 1.38 | 0.51;3.75 |
| **Coagulopathy** | 0 | 1 (2.27%) | 1 (1.56%) | 0.78 | 1.46 | 0.08;24.06 |
| **High blood pressure (hypertension)** | 0 | 16 (36.36%) | 28 (43.75%) | 0.44 | 0.73 | 0.33;1.61 |
| **Chronic obstructive pulmonary disease (COPD)** | 0 | 1 (2.27%) | 6 (9.375%) | 0.14 | 0.47 | 0.01;11.94 |
| **Chronic ischemic heart disease** | 0 | 2 (4.54%) | 3 (4.68%) | 0.97 | 0.47 | 0.01;11.94 |
| **Tobacco smoking** | 0 | 15 (34.09%) | 11 (17.18%) | 0.04 | 2.49 | 1.01;6.13 |
| **Alcohol abuse** | 0 | 4 (9.09%) | 0 (0.00%) | 0.01 | 14.33 | 1.75;273;31 |
| **White Blood Cells (WBC) >15 x 10 3/ul** **(Youden J: 0.57)** | 0 | 23 (52.27%) | 26 (40.62%) | 0.23 | 1.60 | 0.73;3.47 |
| **C-reactive Protein (CRP) >120 mg/l** **(Youden J: 0.76)** | 0 | 25 (56.81%) | 32 (50.00%) | 0.48 | 1.31 | 0.60;2.84 |
| **Creatinine >1.6 mg/dl**  **(Youden J: 0.63)** | 6 | 2 (4.54%) | 4 (6.25%) | 0.79 | 0.79 | 0.13;4.57 |
| **Hemoglobin <13 g/dl**  **(Youden J: 0.84)** | 6 | 19 (48.71%) | 30 (47.61%) | 0.91 | 1.04 | 0.47;2.32 |
| **Platelets < 250 x10 3/ul**  **(Youden J: 0.54)** | 6 | 16 (41.02%) | 24 (38.09%) | 0.76 | 1.13 | 0.50;2.55 |
| **Body temperature >38 ^o^C**  **(Youden J: 0.86)** | 0 | 17 (38.63%) | 32 (50.00%) | 0.24 | 0.63 | 0.28;1.37 |
| **Heart rate >90 bpm**  **(Youden J: 0.72)** | 36 | 15 (44.11%) | 13 (34.21%) | 0.38 | 1.51 | 0.58;3.93 |
| **World Society of Emergency Surgery (WSES) CT scan Ib** | 0 | 2 (4.54%) | 0 (0.00%) | 0.08 | 7.58 | 0.35;161.99 |
| **World Society of Emergency Surgery (WSES) CT scan IIa** | 0 | 42 (95.45%) | 64 (100.00%) | 0.08 | 0.13 | 0.06;2.81 |
| **Presence of air bubbles inside the abscess** | 0 | 23 (52.27%) | 34 (53.12%) | 0.93 | 0.96 | 0.44;2.08 |
| **Previous episodes of diverticulitis >1** | 0 | 3 (6.81%) | 6 (9.37%) | 0.63 | 0.70 | 0.16;2.99 |
| **Hinchey CT scan classification stage IIb** | 0 | 44 (100.00%) | 64 (100.00%) | 0.22 | 0.22 | 0.09;5.64 |
| **Presence of retroperitoneal bubbles** | 0 | 4 (9.09%) | 2 (3.12%) | 0.18 | 3.10 | 0.54;17.72 |
| **Presence of distant free air** | 0 | 10 (22.72%) | 7 (10.93%) | 0.09 | 2.39 | 0.83;6.87 |
| **Presence of free pelvic fluid** | 0 | 11 (25.00%) | 23 (35.93%) | 0.22 | 0.59 | 0.25;1.39 |
| **CT-guided percutaneous drainage** | 0 | 7 (15.90%) | 13 (20.31%) | 0.56 | 0.74 | 0.27;2.04 |
| **Ultrasound-guided percutaneous drainage** | 0 | 6 (13.63%) | 15 (23.43%) | 0.20 | 0.51 | 0.18;1.45 |
| **In-hospital mortality** | 0 | 0 (0.00%) | 1 (1.56%) | 0.40 | 0.47 | 0.01;11.94 |
| **Treatment of the failure: Laparoscopic lavage** | 0 | 3 (6.81%) | NA |  |  |  |
| **Treatment of the failure: Hartmann resection** | 0 | 14 (31.81%) | NA |  |  |  |
| **Treatment of the failure: Colorectal resection with primary anastomosis** | 0 | 25 (56.81%) | NA |  |  |  |
| **Treatment of the failure: Colorectal resection with open abdomen** | 0 | 5 (11.36%) | NA |  |  |  |
| **Time between the beginning of the symptoms and hospital admission >4 days** | 0 | 15 (34.09%) | 19 (29.68%) | 0.62 | 1.22 | 0.53;2.78 |
| **Day of hospital admission (weekend)** | 24 | 9 (22.50%) | 9 (20.45%) | 0.82 | 1.12 | 0.39;3.20 |
| **Symptomatic acute diverticulitis recurrence ≤30 days** | 0 | 0 (0.00%) | 1 (1.56%) | 0.40 | 0.47 | 0.01;11.94 |
| **Symptomatic acute diverticulitis recurrence >30 days** **(to 90-day follow-up**) | 0 | 1 (2.27%) | 6 (9.37%) | 0.14 | 0.22 | 0.02;1.93 |
