# Supplementary material for: Towards a tailored approach for patients with acute diverticulitis and abscess formation. The DivAbsc2023 multicentre case–control study
Source: Surg Endosc. 2024 Apr 17;38(6):3180–94. doi: 10.1007/s00464-024-10793-z (PMC11133057; doi:10.1007/s00464-024-10793-z)
Supplement: Supplementary file 7 — Supplementary file7 (DOC 31 kb) [file 464_2024_10793_MOESM7_ESM.doc]

**Supplementary Table 6.** Results of the multivariable analysis of risk factors for conservative treatment failure (Abscesses >5 cm).

| ***Variable*** | | ***Estimate*** | ***Standard Error*** | ***Adjusted Odds Ratio (aOR)*** | | ***P Value*** | ***95% Confidence Interval (CI)*** |
| --- | --- | --- | --- | --- | --- | --- | --- |
| ***Model 1 Stepwise*** | | | | | | | |
| Age (Years) | | -0.03 | 0.01 | 0.96 | | 0.01 | -0.06;-0.01 |
| Alcohol abuse | | 1.80 | 11.63 | 1.99 | | 0.98 | 2.94;2.56 |
| Model Summary McFadden R^2^ = 0.09 Negelkerke R^2^ = 0.16 Tjur R^2^ = 0.11 Cox&Snell R^2^ = 0.12 | | | | | | | |
| ***Model 2 Stepwise*** | | | | | | | |
| Age (Years) | | -0.03 | 0.01 | 0.96 | | 0.01 | -0.06;-0.01 |
| Alcohol abuse | | 1.71 | 1.97 | 4.95 | | 0.99 | -3.51;3.95 |
| Steroid therapy | | 1.75 | 1.80 | 3.92 | | 0.99 | -3.62;3.51 |
| Model Summary McFadden R^2^ = 0.13 Negelkerke R^2^ = 0.22 Tjur R^2^ = 0.14 Cox&Snell R^2^ = 0.16 | | | | | | | |
| ***Model 3 Stepwise*** | | | | | | | |
| Age (Years) | | -0.03 | 0.01 | 0.96 | | 0.02 | -0.06;-0.01 |
| Alcohol abuse | | 1.78 | 1.94 | 4.32 | | 0.99 | -3.29;3.45 |
| Steroid therapy | | 1.74 | 1.78 | 3.59 | | 0.99 | -3.66;3.37 |
| Tobacco smoking | | 0.85 | 0.50 | 1.35 | | 0.04 | 1.14;1.85 |
| Model Summary McFadden R^2^ = 0.15 Negelkerke R^2^ = 0.25 Tjur R^2^ = 0.17 Cox&Snell R^2^ = 0.18 | | | | | | | |
| ***Model 4 Stepwise*** | | | | | | | |
| Age (Years) | | -0.02 | 0.01 | 0.97 | | 0.12 | -0.05;0.01 |
| Alcohol abuse | | 1.71 | 1.87 | 4.03 | | 0.99 | -3.47;3.50 |
| Steroid therapy | | 1.79 | 1.59 | 3.09 | | 0.99 | -3.49;3.91 |
| Tobacco smoking | | 0.82 | 0.51 | 2.27 | | 0.10 | -0.17;1.82 |
| Diabetes | | 1.55 | 1.21 | 1.39 | | 0.16 | 0.63;3.75 |
| Model Summary McFadden R^2^ = 0.17 Negelkerke R^2^ = 0.28 Tjur R^2^ = 0.19 Cox&Snell R^2^ = 0.20 | | | | | | | |
| ***Model 5 Stepwise*** | | | | | | | |
| Age <40 years | | 1.69 | 0.83 | 1.18 | | 0.04 | 1.05;3.32 |
| Alcohol abuse | | 1.74 | 1.75 | 3.83 | | 0.99 | -3.23;3.16 |
| Steroid therapy | | 1.78 | 1.53 | 3.11 | | 0.99 | -3.42;3.85 |
| Tobacco smoking | | 0.77 | 0.52 | 1.16 | | 0.09 | -0.24;1.79 |
| Diabetes | | 1.82 | 1.07 | 2.17 | | 0.13 | 0.29;3.93 |
| Model Summary McFadden R^2^ = 0.19 Negelkerke R^2^ = 0.30 Tjur R^2^ = 0.21 Cox&Snell R^2^ = 0.22 | | | | | | | |
| ***Model 6 Enter*** | | | | | | | |
| Alcohol abuse | | 1.74 | 1.75 | 3.13 | | 0.99 | -3.23;3.16 |
| Tobacco smoking | | 0.87 | 0.50 | 1.38 | | 0.08 | -0.12;1.86 |
| Age <40 years | | 1.96 | 0.82 | 1.64 | | 0.01 | 1.33;3.58 |
| Model Summary McFadden R^2^ = 0.12 Negelkerke R^2^ = 0.20 Tjur R^2^ = 0.15 Cox&Snell R^2^ = 0.15 | | | | | | | |
| **Accuracy** 0.731 | **AUC** 0.74 | | **Sensitivity** 0.52 | | **Specificity** 0.87 | **Precision** 0.74 | |
