# Supplementary material for: Towards a tailored approach for patients with acute diverticulitis and abscess formation. The DivAbsc2023 multicentre case–control study
Source: Surg Endosc. 2024 Apr 17;38(6):3180–94. doi: 10.1007/s00464-024-10793-z (PMC11133057; doi:10.1007/s00464-024-10793-z)
Supplement: Supplementary file 8 — Supplementary file8 (DOC 117 kb) [file 464_2024_10793_MOESM8_ESM.doc]

**Supplementary Table 7.** Results of the univariable analysis of risk factors for conservative treatment failure (Hinchey IIb stage).

| ***Variable*** | ***Missed data*** | ***Failure*** | ***Success*** | ***P value*** | ***Odds Ratio (OR), or Mean Difference (SE)*** | ***95% Confidence Interval (CI)*** |
| --- | --- | --- | --- | --- | --- | --- |
| ***Failure of conservative treatment*** | *0* | *67 (39.18%)* | *104 (60.81%)* |  |  |  |
| **Age (years)** | 0 | 59.25 ± 14.19 | 63.94 ± 13.82 | 0.03 | 4.68 (2.18) | 0.36;9.00 |
| **Body Mass Index (BMI) (Kg/m^2^)** | 0 | 26.88 ± 4.12 | 26.54 ± 3.95 | 0.59 | -0.33 (0.63) | -1.57;0.91 |
| **Charlson Comorbidity Index** | 0 | 2.09 ± 1.96 | 2.60 ± 3.31 | 0.25 | 0.51 (0.44) | -0.37;1.40 |
| **White Blood Cells (WBC) (x10^3^ u/l)** | 0 | 15.00 ± 4.21 | 13.84 ± 4.70 | 0.10 | -1.16 (0.70) | -2.55;0.23 |
| **C-reactive Protein (CRP) mg/l** | 0 | 128.09 ± 75.02 | 127.73 ± 93.97 | 0.97 | -0.36 (13.63) | -27.28;26.56 |
| **Creatinine (mg/dl)** | 18 | 1.01 ± 1.05 | 1.05 ± 0.59 | 0.66 | 0.03 (0.08) | -0.13;0.21 |
| **Hemoglobin (g/dl)** | 18 | 13.09 ± 1.83 | 13.18 ± 1.65 | 0.76 | 0.08 (0.28) | -0.48;0.65 |
| **Platelets (x10^3^ u/l)** | 19 | 289.87 ± 117.26 | 281.30 ± 99.11 | 0.36 | -8.57 (17.80) | -43.74;26.60 |
| **Procalcitonin (ng/ml)** | 133 | 1.12 ± 1.18 | 6.25 ± 21.03 | 0.40 | 5.12 (6.12) | -7.28;17.53 |
| **Body temperature (^o^C)** | 0 | 37.57 ± 0.86 | 37.41 ± 0.92 | 0.26 | -0.15 (0.14) | -0.43;0.12 |
| **Systolic blood pressure (mmHg)** | 60 | 137.87 ± 18.12 | 129.88 ± 19.30 | 0.02 | -7.99 (3.59) | -15.11;-0.87 |
| **Heart rate (bpm)** | 63 | 91.66 ± 16.28 | 86.13 ± 18.09 | 0.10 | -5.33 (3.35) | -12.18;1.11 |
| **Abscess diameter on CT scan (mm)** | 0 | 66.46 ± 29.33 | 63.43 ± 22.05 | 0.45 | -3.03 (4.02) | -10.98;4.90 |
| **Length of antibiotic therapy (days)** | 96 | 8.81 ± 5.28 | 9.18 ± 3.29 | 0.38 | 0.87 (0.99) | -1.10;2.85 |
| **Time between the beginning of symptoms and hospital admission (days)** | 0 | 3.82 ± 3.10 | 4.13 ± 3.63 | 0.56 | 0.31 (0.53) | -0.74;1.37 |
| **Time spent in the Emergency Department (minutes)** | 62 | 338.00 (IQR 292.50) | 307.00 (IQR 340.0) | 0.18 | 28.00 (222.96) | -59.00;119.00 |
| **Length of hospital stay (days)** | 0 | 17.19 ± 10.48 | 11.19 ± 6.31 | <0.01 | -6.00 (1.28) | -8.54;-3.46 |
| **Previous episodes of acute diverticulitis** | 0 | No previous episodes  45 (67.16%) | No previous episodes  93 (89.42%) | 0.55 | -0.14 (0.23) | -0.60;0.32 |
|  |  | 1 previous episode  16 (23.88%) | 1 previous episode  10 (9.61%) |  |  |  |
|  |  | > 1 previous episode  6 (8.95%) | > 1 previous episode  1 (0.96%) |  |  |  |
| **Number of abscesses on CT scan** | 0 | 1 abscess  61 (91.04%) | 1 abscess  93 (89.42%) | 0.87 | -0.02 (0.14) | -0.31;0.26 |
|  |  | 2 abscesses  6 (8.95%) | 2 abscesses  10 (9.61%) |  |  |  |
|  |  | >2 abscesses  0 (0.00%) | >2 abscesses  1 (0.96%) |  |  |  |
| **Air bubbles inside the abscess** | 0 | 0 bubbles  32 (47.76%) | 0 bubbles  51 (49.03%) | 0.98 | -0.01 (0.17) | -0.35;0.34 |
|  |  | 1 bubble  18 (26.86%) | 1 bubble  25 (24.03%) |  |  |  |
|  |  | >1 bubble  17 (25.37%) | >1 bubble  28 (26.92%) |  |  |  |
| **Time of hospital admission** | 34 | 06.01-12.00  22 (36.66%) | 06.01-12.00  21 (27.27%) | 0.07 | NA | NA |
|  |  | 12.01-18.00  18 (30.00%) | 12.01-18.00  16 (20.77%) |  |  |  |
|  |  | 18.01-23.59  11 (18.33%) | 18.01-23.59  30 (38.96%) |  |  |  |
|  |  | 00.00-06.00  9 (15.00%) | 00.00-06.00  10 (12.97%) |  |  |  |
| **In-hospital morbidity (Clavien-Dindo)** | 0 | No morbidity  38 (56.71%) | No morbidity  99 (95.19%) | <0.01 | 2.32 (0.33) | 1.66;2.99 |
|  |  | Clavien-Dindo 1  9 (13.43%) | Clavien-Dindo 1  2 (1.92%) |  |  |  |
|  |  | Clavien-Dindo 2  11 (16.41%) | Clavien-Dindo 2  1 (0.96%) |  |  |  |
|  |  | Clavien-Dindo 3a  2 (2.98%) | Clavien-Dindo 3a  2 (1.92%) |  |  |  |
|  |  | Clavien-Dindo 3b  6 (8.95%) | Clavien-Dindo 3b  0 (0.00%) |  |  |  |
|  |  | Clavien-Dindo 4a  1 (1.49%) | Clavien-Dindo 4a  0 (0.00%) |  |  |  |
|  |  | Clavien-Dindo 4b  0 (0.00%) | Clavien-Dindo 4b  0 (0.00%) |  |  |  |
| **Diverticulitis recurrence characteristics** | 0 | Obstruction  0 (0.00%) | Obstruction  1 (0.96%) | 0.27 | NA | NA |
|  |  | Abscess  0 (0.00%) | Abscess  8 (7.69%) |  |  |  |
|  |  | Perforation  2 (2.98%) | Perforation  6 (5.76%) |  |  |  |
| **Age >60 years** **(Youden J: 0.48)** | 0 | 36 (53.73%) | 66 (63.46%) | 0.20 | 0.66 | 0.35;1.24 |
| **Female gender** | 0 | 33 (49.25%) | 50 (48.07%) | 0.88 | 1.04 | 0.56;1.93 |
| **Body Mass Index (BMI) >28 Kg/m ^2^**  **(Youden J: 0.53)** | 0 | 20 (29.85%) | 32 (30.76%) | 0.89 | 0.95 | 0.49;1.86 |
| **Charlson Comorbidity Index >3**  **(Youden J: 0.45)** | 0 | 13 (19.40%) | 30 (28.84%) | 0.16 | 0.59 | 0.28;1.24 |
| **Immunodeficiency (Congenital/Acquired)** | 0 | 0 (0.00%) | 2 (1.92%) | 0.25 | 0.30 | 0.01;6.42 |
| **Diabetes** | 0 | 5 (7.46%) | 15 (4.42%) | 0.16 | 0.47 | 0.16;1.38 |
| **Chronic Kidney Disease** | 0 | 2 (2.98%) | 4 (3.84%) | 0.76 | 0.76 | 0.13;4.32 |
| **Dialysis** | 0 | 1 (1.49%) | 0 (0.00%) | 0.21 | 4.71 | 0.18;117.44 |
| **Active tumor** | 0 | 1 (1.49%) | 2 (1.92%) | 0.83 | 0.77 | 0.06;8.69 |
| **Steroid therapy** | 0 | 2 (2.98%) | 7 (6.73%) | 0.28 | 0.42 | 0.08;2.11 |
| **Chemotherapy** | 0 | 1 (1.49%) | 0 (0.00%) | 0.21 | 4.71 | 0.18;117.44 |
| **Immunotherapy** | 0 | 0 (0.00%) | 1 (0.96%) | 0.42 | 0.51 | 0.02;12.73 |
| **Chronic cardiac failure** | 0 | 2 (2.98%) | 3 (2.88%) | 0.97 | 1.03 | 0.16;6.36 |
| **Chronic pulmonary failure** | 0 | 1 (1.49%) | 2 (1.92%) | 0.83 | 0.77 | 0.06;8.69 |
| **Obesity** | 0 | 17 (25.37%) | 19 (18.26%) | 0.26 | 1.52 | 0.72;3.19 |
| **Coagulopathy** | 0 | 1 (1.49%) | 2 (1.92%) | 0.83 | 0.77 | 0.06;8.69 |
| **High blood pressure (hypertension)** | 0 | 27 (40.29%) | 43 (41.34%) | 0.89 | 0.95 | 0.51;1.78 |
| **Chronic obstructive pulmonary disease (COPD)** | 0 | 4 (5.97%) | 7 (6.73%) | 0.84 | 0.88 | 0.24;3.12 |
| **Chronic ischemic heart disease** | 0 | 2 (2.98%) | 5 (4.80%) | 0.55 | 0.60 | 0.11;3.23 |
| **Tobacco smoking** | 0 | 23 (34.32%) | 21 (20.19%) | 0.03 | 2.06 | 1.03;4.14 |
| **Alcohol abuse** | 0 | 5 (7.46%) | 3 (2.88%) | 0.16 | 2.71 | 0.62;11.76 |
| **White Blood Cells (WBC) >15 x10 3/ul**  **(Youden J: 0.75)** | 0 | 32 (47.76%) | 39 (37.50%) | 0.18 | 1.52 | 0.81;2.84 |
| **C-reactive Protein (CRP) >120 mg/l**  **(Youden J: 0.74)** | 0 | 33 (49.25%) | 45 (43.26%) | 0.44 | 1.27 | 0.68;2.35 |
| **Creatinine >1.6 mg/dl** **(Youden J: 0.53)** | 18 | 4 (6.89%) | 5 (5.26%) | 0.67 | 1.33 | 0.34;5.18 |
| **Hemoglobin <13 g/dl** **(Youden J: 0.81)** | 18 | 28 (48.27%) | 42 (44.21%) | 0.62 | 1.17 | 0.61;2.26 |
| **Platelets < 250 x10 3/ul** **(Youden J: 0.63)** | 19 | 27 (47.36%) | 42 (44.21%) | 0.70 | 1.13 | 0.58;2.19 |
| **Body temperature >38 ^o^C** **(Youden J: 0.65)** | 0 | 28 (41.79%) | 52 (50.00%) | 0.29 | 0.71 | 0.38;1.33 |
| **Heart rate >90 bpm** **(Youden J: 0.46)** | 63 | 20 (41.66%) | 20 (33.33%) | 0.37 | 1.42 | 0.65;3.13 |
| **Abscess diameter >5 cm** | 0 | 43 (64.17%) | 65 (62.50%) | 0.78 | 1.09 | 0.57;2.06 |
| **World Society of Emergency Surgery (WSES) CT scan IIa** | 0 | 66 (98.50%) | 101 (97.11%) | 0.55 | 1.96 | 0.20;19.24 |
| **Presence of air bubbles inside the abscess** | 0 | 33 (49.25%) | 52 (50.00%) | 0.92 | 0.97 | 0.52;1.79 |
| **Previous episodes of diverticulitis >1** | 0 | 6 (8.95%) | 10 (9.61%) | 0.88 | 0.92 | 0.32;2.67 |
| **Presence of retroperitoneal bubbles** | 0 | 5 (7.46%) | 4 (3.84%) | 0.30 | 2.01 | 0.52;7.79 |
| **Presence of distant free air** | 0 | 13 (19.40%) | 9 (8.65%) | 0.04 | 2.54 | 1.02;6.33 |
| **Presence of free pelvic fluid** | 0 | 18 (26.86%) | 36 (34.61%) | 0.28 | 0.69 | 0.35;1.36 |
| **CT-guided percutaneous drainage** | 0 | 8 (11.94%) | 16 (15.38%) | 0.52 | 0.74 | 0.30;1.85 |
| **Ultrasound-guided percutaneous drainage** | 0 | 6 (8.95%) | 17 (16.34%) | 0.16 | 0.50 | 0.18;1.35 |
| **In-hospital mortality** | 0 | 1 (1.49%) | 1 (0.96%) | 0.75 | 1.56 | 0.09;25.38 |
| **Treatment of the failure: Laparoscopic lavage** | 0 | 10 (14.92%) | NA | NA | NA | NA |
| **Treatment of the failure: Hartmann resection** | 0 | 23 (34.32%) | NA | NA | NA | NA |
| **Treatment of the failure: Colorectal resection with primary anastomosis** | 0 | 35 (52.23%) | NA | NA | NA | NA |
| **Treatment of the failure: Colorectal resection with open abdomen** | 0 | 7 (10.44%) | NA | NA | NA | NA |
| **Time between the beginning of the symptoms and hospital admission >4 days** | 0 | 17 (25.37%) | 27 (25.97%) | 0.93 | 0.97 | 0.48;1.96 |
| **Day of hospital admission (weekend)** | 33 | 10 (16.39%) | 14 (18.18%) | 0.78 | 0.88 | 0.36;2.15 |
| **Symptomatic acute diverticulitis recurrence ≤30 days** | 0 | 1 (1.49%) | 2 (1.92%) | 0.83 | 0.77 | 0.06;8.69 |
| **Symptomatic acute diverticulitis recurrence >30 days (to 90-day follow-up**) | 0 | 1 (1.49%) | 13 (12.50%) | 0.01 | 0.10 | 0.01;0.83 |
