# Supplementary material for: Towards a tailored approach for patients with acute diverticulitis and abscess formation. The DivAbsc2023 multicentre case–control study
Source: Surg Endosc. 2024 Apr 17;38(6):3180–94. doi: 10.1007/s00464-024-10793-z (PMC11133057; doi:10.1007/s00464-024-10793-z)
Supplement: Supplementary file 9 — Supplementary file9 (DOC 29 kb) [file 464_2024_10793_MOESM9_ESM.doc]

**Supplementary Table 8.** Results of the multivariable analysis of risk factors for conservative treatment failure (Hinchey IIb stage).

| ***Variable*** | ***Estimate*** | ***Standard Error*** | | ***Adjusted Odds Ratio (aOR)*** | ***P Value*** | ***95% Confidence Interval (CI)*** |
| --- | --- | --- | --- | --- | --- | --- |
| ***Model 1 Stepwise*** | | | | | | |
| Tobacco smoking | 1.07 | 0.45 | | 2.91 | 0.01 | 1.19;7.11 |
| Presence of distant free air | 1.04 | 0.53 | | 2.84 | 0.05 | 0.99;8.12 |
| Model Summary McFadden R^2^ = 0.06 Negelkerke R^2^ = 0.10 Tjur R^2^ = 0.08 Cox&Snell R^2^ = 0.07 | | | | | | |
| ***Model 2 Stepwise*** | | | | | | |
| Tobacco smoking | 1.07 | 0.46 | | 2.93 | 0.02 | 1.18;7.22 |
| Presence of distant free air | 0.95 | 0.54 | | 2.58 | 0.08 | 0.89;7.48 |
| Age (Years) | -0.02 | 0.01 | | 0.97 | 0.09 | 0.94;1.00 |
| Model Summary McFadden R^2^ = 0.07 Negelkerke R^2^ = 0.13 Tjur R^2^ = 0.10 Cox&Snell R^2^ = 0.10 | | | | | | |
| ***Model 3 Stepwise*** | | | | | | |
| Tobacco smoking | 0.93 | 0.47 | | 2.55 | 0.04 | 1.00;6.46 |
| Presence of distant free air | 0.72 | 0.56 | | 2.07 | 0.19 | 0.68;6.28 |
| Age (Years) | -0.03 | 0.01 | | 0.96 | 0.03 | 0.93;0.99 |
| Systolic blood pressure (mmHg) | 0.02 | 0.01 | | 1.02 | 0.04 | 1.00;1.04 |
| Model Summary McFadden R^2^ = 0.10 Negelkerke R^2^ = 0.18 Tjur R^2^ = 0.13 Cox&Snell R^2^ = 0.13 | | | | | | |
| ***Model 4 Stepwise*** | | | | | | |
| Tobacco smoking | 0.85 | 0.46 | | 2.35 | 0.06 | 0.94;5.88 |
| Age (Years) | -0.03 | 0.01 | | 0.96 | 0.01 | 0.93;0.99 |
| Systolic blood pressure (mmHg) | 0.02 | 0.01 | | 1.02 | 0.02 | 1.00;1.05 |
| Model Summary McFadden R^2^ = 0.09 Negelkerke R^2^ = 0.16 Tjur R^2^ = 0.12 Cox&Snell R^2^ = 0.12 | | | | | | |
| ***Model 5 Stepwise*** | | | | | | |
| Age <40 (Years) | 1.21 | 0.63 | | 1.29 | 0.02 | 1.07;1.85 |
| Tobacco smoking | 0.92 | 0.45 | | 2.75 | 0.04 | 1.10;6.47 |
| Model Summary McFadden R^2^ = 0.04 Negelkerke R^2^ = 0.07 Tjur R^2^ = 0.05 Cox&Snell R^2^ = 0.05 | | | | | | |
| ***Model 6 Enter*** | | | | | | |
| Tobacco smoking | 0.93 | 0.47 | 2.55 | | 0.04 | 1.01;6.46 |
| Presence of distant free air | 0.72 | 0.56 | 2.07 | | 0.19 | 0.68;6.28 |
| Age (Years) | -0.03 | 0.01 | 0.96 | | 0.03 | 0.93;0.99 |
| Systolic blood pressure | 0.02 | 0.01 | 1.02 | | 0.04 | 1.01;1.04 |
| Model Summary McFadden R^2^ = 0.10 Negelkerke R^2^ = 0.18 Tjur R^2^ = 0.13 Cox&Snell R^2^ = 0.13 | | | | | | |
| **Accuracy** 0.68 | **AUC** 0.71 | **Sensitivity** 0.59 | **Specificity** 0.75 | | **Precision** 0.65 | |
